# Using Haplotype-Based Artificial Intelligence to Evaluate SARS-CoV-2 Novel Variants and Mutations

**DOI:** 10.1001/jamanetworkopen.2023.0191

**Published:** 2023-02-21

**Authors:** Lue Ping Zhao, Seth Cohen, Michael Zhao, Margaret Madeleine, Thomas H. Payne, Terry P. Lybrand, Daniel E. Geraghty, Keith R. Jerome, Lawrence Corey

**Affiliations:** 1 Public Health Sciences Division, Fred Hutchinson Cancer Research Center, Seattle, Washington; 2 Vaccine and Infectious Disease Division, Fred Hutchinson Cancer Research Center, Seattle, Washington; 3 Department of Medicine, University of Washington School of Medicine, Seattle; 4 QuantFu Inc, Boston, Massachusetts; 5 Quintepa Computing LLC, Nashville, Tennessee; 6 Department of Chemistry, Vanderbilt University; Nashville, Tennessee; 7 Clinical Research Division, Fred Hutchinson Cancer Research Center, Seattle, Washington

## Abstract

**Importance:**

Earlier detection of emerging novel SARS-COV-2 variants is important for public health surveillance of potential viral threats and for earlier prevention research. Artificial intelligence may facilitate early detection of SARS-CoV2 emerging novel variants based on variant-specific mutation haplotypes and, in turn, be associated with enhanced implementation of risk-stratified public health prevention strategies.

**Objective:**

To develop a haplotype-based artificial intelligence (HAI) model for identifying novel variants, including mixture variants (MVs) of known variants and new variants with novel mutations.

**Design, Setting, and Participants:**

This cross-sectional study used serially observed viral genomic sequences globally (prior to March 14, 2022) to train and validate the HAI model and used it to identify variants arising from a prospective set of viruses from March 15 to May 18, 2022.

**Main Outcomes and Measures:**

Viral sequences, collection dates, and locations were subjected to statistical learning analysis to estimate variant-specific core mutations and haplotype frequencies, which were then used to construct an HAI model to identify novel variants.

**Results:**

Through training on more than 5 million viral sequences, an HAI model was built, and its identification performance was validated on an independent validation set of more than 5 million viruses. Its identification performance was assessed on a prospective set of 344 901 viruses. In addition to achieving an accuracy of 92.8% (95% CI within 0.1%), the HAI model identified 4 Omicron MVs (Omicron-Alpha, Omicron-Delta, Omicron-Epsilon, and Omicron-Zeta), 2 Delta MVs (Delta-Kappa and Delta-Zeta), and 1 Alpha-Epsilon MV, among which Omicron-Epsilon MVs were most frequent (609/657 MVs [92.7%]). Furthermore, the HAI model found that 1699 Omicron viruses had unidentifiable variants given that these variants acquired novel mutations. Lastly, 524 variant-unassigned and variant-unidentifiable viruses carried 16 novel mutations, 8 of which were increasing in prevalence percentages as of May 2022.

**Conclusions and Relevance:**

In this cross-sectional study, an HAI model found SARS-COV-2 viruses with MV or novel mutations in the global population, which may require closer examination and monitoring. These results suggest that HAI may complement phylogenic variant assignment, providing additional insights into emerging novel variants in the population.

## Key Points

**Question:**

Could viral genetic mutations and associated haplotypes be used to identify emerging novel SARS-COV-2 variants?

**Findings:**

In this cross-sectional study, a haplotype-based artificial intelligence (HAI) model was trained on more than 5 million viral sequences to identify emerging novel SARS-COV-2 variants due to the acquisition of new mutations or mixture of mutations from multiple variants. Applying HAI to 344 901 viral sequences identified 7 mixture variants (eg, Omicron-Alpha, Omicron-Epsilon, Omicron-Zeta, and Alpha-Epsilon) and 16 novel mutations, 8 of which were increasing in prevalence percentage in the earlier part of May 2022.

**Meaning:**

The successful application of HAI in this study suggests its utility in identifying novel emerging SARS-COV-2 variants even if such variants have not been observed previously.

## Teaser

This cross-sectional study investigates the use of haplotype-based artificial intelligence to identify novel SARS-COV-2 variants and mutations.

## Introduction

The COVID-19 pandemic is gradually shifting to an endemic phase with continuously circulating SARS-COV-2 variants globally. The presence of multiple viral variants increases co-infection risks, which may lead to recombinants (eg, an Alpha-Omicron mixture) as new, emerging variants.^1,2,3,4,5^ In addition, every infection could recombine with mutations from other viruses,^3^ host genetic sequences,^6^ or zoonotic events,^7^ with saltational outcomes that may lead to new variants. Most mutations are functionally neutral, appearing and waning randomly, but some may persist because they impart increased transmissibility or virulence. Therefore, detecting new variants is of importance, for example, to facilitate early viral control measures and enhanced lead time to research and develop effective preventive and treatment strategies.

Prevailing methods of identifying variants assign sequenced viruses to known clades and lineages using phylogenic methods.^8,9,10,11^ When a group of viral clades or lineages emerges rapidly and exhibits excessive transmissibility, virulence, or evasion of host immunity, these variants are classified as variants of interest and variants of concern by an expert panel of the World Health Organization (WHO)^12^ and are classified further as variants being monitored or variants of high consequence by the US Centers for Disease Control and Prevention (CDC).^13^ Currently, phylogenic methods^14,15,16^ are routinely applied in classifying all viruses, and assigned lineages and clades are accepted by WHO^17^ and the CDC^13^ to identify variants and declare the emergence of new variants. However, such variant assignments may be uncertain when multiple variants are recombined and the assumption of branching phylogenic trees, required by most phylogenic methods in use, is violated. Ignoring this violation could bias phylogenetic inferences.^18,19^ When applied to classifying SARS-COV-2, conventional phylogenic analysis may force assignment of a recombinant variant to an existing variant (ie, a misclassification error) or may miss the recombinant variant (ie, a missing data error).

There are alternative approaches for identifying mutations in SARS-COV-2. One approach is to estimate mutational drivers of individual genes based on amino acid substitutions in individual SARS-COV-2 genes.^20^ Another approach is an empirical statistical learning strategy (SLS) that selects individual polymorphic amino acid sites (hereafter, *polymutants*), models their temporal patterns over time, and identifies haplotypes based on a set of polymutants that share synchronized expansion patterns.^21^ The primary limitation of these 2 alternative approaches is the lack of direct linkage of specific mutations or polymutants with variant assignments, which makes interpretation difficult.

Using existing analytic approaches and the large viral sequence database at Global Initiative on Sharing Avian Influenza Data (GISAID),^22,23,24^ we sought to build a haplotype-based artificial intelligence (HAI) model for identifying SARS-COV-2 novel variants using variant-specific polymutants and their haplotypes. In addition to identifying variants, the HAI model was designed to discover novel variants with no need for the branching phylogenic trees assumption. Conceptually, the HAI model learned from the large collection of viral sequences in GISAID to identify core polymutants that were specific to viral variants. Through a haplotype analysis, the HAI model estimated haplotype frequencies of variant-specific core polymutant haplotypes. Applying Bayes’ theorem, HAI computed identification probabilities corresponding to all known variants. By a chosen threshold probability, estimated variant identification probabilities were used to identify the variant under which each virus should be classified, including variant-unidentifiable viruses with novel mutations. If variant identification was ambiguous, with 2 or more identification probabilities greater than a prespecified threshold, the result implied that the viral genome had appreciable probabilities of carrying corresponding variant-specific core haplotypes (ie, a mixture of corresponding variants), possibly due to recombination. From GISAID, we obtained 10.5 million viral sequences (downloaded on March 14, 2022), with half as training set and the rest as a validation set, to develop and validate the HAI model. To demonstrate its identification performance, we used pooled data to build the final HAI model and applied it to a prospective set of 344 901 viruses collected from March 15 to May 18, 2022. Using identification results from the prospective set, we explored mixture variants (MVs) and viruses with novel mutations to gain insights into emerging SARS-COV-2 variants.

## Methods

Because GISAID data may be considered as observational routinely collected health data, they are reported following the Reporting of Studies Conducted Using Observational Routinely-collected Health Data (RECORD) guideline.^22^ This study was determined to be exempt from review by Fred Hutchinson Research Center institution review board and informed consent was waived because the identity of the human participants cannot readily by ascertained directly or through identifiers linked to the participants, in accordance with 45 CFR §46.104(d)(4).

### GISAID and Study Design

GISAID is a central data portal for storing genomic sequences for coronaviruses in the COVID-19 pandemic.^23,25,26^ Given the large sample size and rapid accumulation of viral sequences at GISAID, we designed this study in 2 phases. The first phase was to train and validate an HAI model, while the second phase was to assess the performance of HAI on a prospectively collected set of viruses.

Accessing GISAID on March 14, 2022, we retrieved all available samples collected between January 1, 2020, and March 14, 2022 (10 450 718 samples). We filtered out samples if viral sequences had fewer than 27 000 nucleotides (119 277 samples [1.1%]), collection dates were incomplete (290 917 samples [2.8%]), or collection dates were prior to January 1, 2020 (33 samples [0.01%]), netting a total of 10 051 620 viruses for this development. By random sampling, half were selected into the training set and the rest into the validation set. For the second-phase analysis, we retrieved samples collected by May 18, 2022; excluded samples collected prior to March 14, 2022; and retained 344 901 viruses in the prospective data set.

### Viral Polymutants

GISAID aligns submitted viral sequences, translates these to amino acids, assigns lineages, extracts mutations (substituting mutations, insertions, and deletions), and disseminates assigned lineages, clades, variants, and sequence mutating amino acids through patient-specific metadata. Mutating amino acids, if they have 3 or more observations, are extracted as viral polymutants to be analyzed. Multiple polymutants from a single virus form a polymutant haplotype because an RNA virus is single stranded. As of May 18 2022, there were 14 variants officially assigned at GISAID (eTable 1 in Supplement 1).

### Sample Collection Location and Date

Metadata included sample collection location and date. The location was organized by continent, country, region, and subregion and had no missing data. A fraction of collection dates were missing completely or partially. Location and date information allowed geographic and temporal analysis of polymutant haplotypes.

### Statistical Analysis

We applied an SLS to develop an HAI model, details of which are provided in eMethods in Supplement 1. Briefly, the SLS included a generalized additive model that was used to select variant-specific polymutants, a haplotype analysis to estimate frequencies of core haplotypes within each variant, a Bayes probabilities to estimate variant-specific posterior probabilities, and an unsupervised learning technique to organize temporal patterns.

## Results

### Association of Polymutant Haplotypes With Viral Variants

SARS-COV-2 viruses are classified in clades and lineages by GISAID based on whole viral genome sequences^27^ and are assigned to variants by GISAID (eTable 1 in Supplement 1). Characteristically, each variant has a group of amino acid substitutions (ie, variant-specific polymutants). To identify such polymutants, we used the training set and extracted polymorphic amino acids from viruses of a specific variant. By comparing observed amino acids against their references, SLS recognized whether amino acids are substitutions and created a binary mutation indicator of 1 or 0, respectively. Associating mutation indicators with collection dates via a generalized additive model, SLS modeled temporal expansions of individual amino acids, based on which locally averaged mutation percentages (LAMP) over time (see eFigure 1 in Supplement 1 for variant-specific expansions) were estimated along with a *P* value quantifying whether temporal trends were significant. We considered a substitution as a variant-specific polymutant if its *P* value was less than. 05 and its maximum LAMP at any time exceeded 10% or if the mean LAMP was greater than 0.5. For all SARS-COV-2 variants (Alpha, Beta, Delta, Epsilon, Eta, Gamma, GH/490R, Iota, Kappa, Lambda, Mu, Omicron, Theta, and Zeta), SLS identified 19, 20, 33, 14, 14, 21, 24, 21, 25, 21, 32, 63, 26, and 10 polymutants, respectively (eTable 2-15 in Supplement 1). Using viral sequences, SLS performed a haplotype analysis to estimate haplotype frequencies, referred to as frequencies of core variant haplotypes (listed in eTables 2-15 in Supplement 1). Empirically, proportions of SARS-COV-2 variants in the general population were estimated in the training set, denoted as *f*(variant = *v*).

By the Bayes theorem, HAI computes probabilities of observing a variant *v*, given viral genome (ie, polymutant haplotype), via the following formula:

*p*(Variant = *v*|*h*) = [*f* (*h*|Variant = *v*)*f* (Variant = *v*)]/*f*(*h*|Unassigned)*f*(Unassigned) + Σ*_v_* *f*(*h*|*v*)*f*(*v*)

in which the summation Σ*_v_* is over all 14 known variants, haplotype frequency *f* (h | variant = *v*) and variant proportion *f (*variant = *v*) are empirically estimated from the training set, in addition to *f* (*h* | Unassigned) and *f* (Unassigned) for variant-unassigned viruses. For each viral sequence, HAI computed an array of variant probabilities. Given the threshold value *p*_v_ = 0.99 for classifying a variant, HAI classified a virus to variant *v* if the corresponding probability was greater than *p*_v_. On the training set, we tabulated concordances of HAI classifications and GISAID assigned variants, which are displayed as a 16 by 15 contingency table (eTable 16 in Supplement 1), that is, 14 known variants and an unassigned virus by GISAID, and 14 identified known variants, 1 unidentifiable variant, and MVs that may be recombinants. For all 5 025 810 virus sequences, the concordance rate of HAI and GISAID variant assignments was 4 326 921 sequences (86.1%), while the discordance rate was nearly zero (5026 sequences [<0.1%]) (Table 1). Among 543 402 unassigned viruses, 175 434 viruses (3.5%) viruses were assigned to 1 known variant and 7633 viruses (0.2%)that were not assigned variants were identified as MVs. Meanwhile, for 4 482 408 viruses with assigned variants, 159 272 viruses (3.6%) were identified as MVs and 7633 viruses (0.1%) were deemed unidentifiable. Finally, 360 335 viruses (7.2%) received no variant assignment by GISAID or identification by HAI. Note that we profiled the choice of threshold value *p*_v_ from 0.90 to 1.00 and found that the choice of 0.99 was associated with a minimum number of 53 discordances (eFigure 2 in Supplement 1). Additionally, note that use of concordance and discordance was suboptimal given that identified variants were not present in the training set.

**Table 1.** Performance Statistics of Haplotype-Based Artificial Intelligence

Performance statistic	%			
	Training	Validation	Full	Prospective
Sample size, No.	5 025 810	5 025 810	10 051 620	344 901
Concordance of identified and assigned variants	86.094	86.334	86.384	92.776
Discordance of identified and assigned variants	0.001	0.040	0.002	0.003
Identified variants of unassigned viruses	3.491	3.487	3.599	0.166
Identified mixtures of unassigned viruses	0.152	0.155	0.179	0.009
Identified mixtures of known variants	3.017	2.511	2.728	6.401
Unidentified variants				
Of known variants	0.075	0.307	0.077	0.494
And unassigned viruses	7.170	7.165	7.031	0.152

### Independent Validation of Haplotype-Based Identifications

Using the same data-processing protocol, we extracted all variant-specific core haplotypes of selected polymutants in the validation set. Using estimated haplotype frequencies and variant proportions, we computed the variant identification probability by the previously described equation. With the chosen threshold, we identified the virus to be a known variant, a mixture of known variants, or an unidentifiable variant. Comparing identifiable variants (by rows) against variant assignment (by columns) by GISAID, we tabulated their concordances and discordances (Table 2). Concordant assignments of known variants by HAI and GISAID are shown along the diagonal line. Results from the concordance analysis in the validation data set were comparable to those in the training data set (Table 1). For example, the estimated concordance between identified and assigned variants was 86.1% and 86.3% in training and validation sets, respectively. We evaluated the concordance between HAI and GISAID, which was measured by a κ statistic,^28^ measuring concordances between GISAID assignments and HAI identifications of 14 known variants, yielding a κ value of nearly 1.00. The κ value, after including unassigned and mixture or unidentifiable viruses, was 0.91.

**Table 2.** Concordance Analysis in Independent Validation Data Set

HAI variant identification						GISAID variant assignment, No.											
	Alpha	Beta	Delta	Epsilon	Eta		Gamma	GH/490R	Iota	Kappa	Lambda	Mu	Omicron	Theta	Zeta	UA	Subtotal
Alpha	574 378	1	27	0	0		3	0	2	0	0	0	6	0	0	2481	576 898
Beta	0	8264	0	0	0		0	0	0	0	0	0	0	0	0	85	8349
Delta	6	2	2 118 401	0	0		2	0	0	12	0	0	98	0	0	1405	2 119 926
Epsilon	93	562	62	36 019	20		26	4	311	1	0	68	20	1	0	145 890	183 077
Eta	1	1	8	0	4711		0	0	0	0	0	0	1	0	0	42	4764
Gamma	3	0	0	0	0		59 327	0	0	0	0	0	0	0	0	96	59 426
GH/490R	0	0	0	0	0		0	446	0	0	0	0	0	0	0	1	447
Iota	2	0	0	0	0		0	0	18 527	0	0	0	0	0	0	65	18 594
Kappa	0	0	601	0	0		0	0	0	3666	0	0	3	0	0	49	4319
Lambda	0	0	0	0	0		0	0	0	0	4970	0	0	0	0	8	4978
Mu	0	0	1	0	0		0	0	0	0	0	6028	0	0	0	31	6060
Omicron	0	0	22	0	0		0	0	0	0	1	0	1 501 145	0	0	642	1 501 810
Theta	0	0	0	0	0		0	0	0	0	0	0	0	115	0	0	115
Zeta	1	0	5	0	0		34	0	0	0	0	0	11	4	2967	24 475	27 497
MV	234	11 982	19 019	17	4		709	2	2681	52	5	1885	89 449	175	1	7794	134 009
UP	474	109	5014	56	6		104	4	24	116	19	65	9445			360 105	375 541
Subtotal	575 192	20 921	2 143 160	36 092	4741		60 205	456	21 545	3847	4995	8046	1 600 178	295	2968	543 169	5 025 810

Abbreviations: GISAID, Global Initiative on Sharing Avian Influenza Data; HAI, haplotype-based artificial intelligence; MV, mixture of variants; UA unassigned variant; UP, unidentifiable variant.

### Identifying Viral Variants Prospectively

The successful validation suggested that HAI-identified variants were highly concordant with GISAID assignments. Integrated variant assignment and identification provided additional insights into emerging novel variants. To evaluate practical utility, we pooled training and validation sets to build a final HAI model with 10 051 620 viral sequences and repeated the same SLS process, except estimating variant proportions with viruses collected from March 15, 2021, to March 14, 2022. The concordance analysis of HAI and GISAID variant assignment on the full data set is shown in eTable 17 in Supplement 1, and estimated concordance and discordance rates were comparable to training set results (Table 1).

Applying the final HAI model to 344 901 prospectively collected viruses, we found that the most common variant was Omicron (343 592 viruses [99.6%]), while there were 2 Alpha, 180 Delta, and 1 Lambda variant viruses (eTable 18 in Supplement 1; Table 3); 1126 viruses were not assigned to any variants. HAI, on the other hand, identified additional variants (Epsilon, Eta, and Zeta) and 2227 MVs (eTable 18 in Supplement 1). To assess which MVs were likely recombinants, we applied a postidentification procedure (eMethods in Supplement 1) under the assumption that if a mixture was from recombination, it must include unique core polymutants to the corresponding variants in the mixture. Most MVs had only Omicron polymutants (647 of 657 variants [98.5%]) (Table 3), and no MVs had polymutants from 3 or more variants; the remaining MVS were classified as 1 of 7 specific MVs (3 Delta-Kappa, 2 Delta-Zeta, 10 Alpha-Epsilon, 25 Omicron-Alpha, 3 Omicron-Delta, 609 Omicron-Epsilon, and 10 Omicron-Zeta MVs). Finally, the HAI model left 2227 viruses unidentified, which included 4 Delta and 1699 Omicron variants. Concordance and discordance rates were 92.776% (95% CI, 92.775%-92.777%) and 0.004% (95% CI, 0.003%-0.005%), respectively (Table 1). Through a formal concordance κ analysis, the κ value for known variants was estimated at 0.96 (95% CI, 0.97-1.00).

**Table 3.** Concordance Analysis in Prospective Data Set^a^

HAI variant identification^b^	GISAID variant assignment, No.								
	Alpha	Delta	Epsilon	Eta	Lambda	Omicron	Zeta	UA	Subtotal
Alpha	2	0	0	0	0	4	0	0	6
Delta	0	171	0	0	0	3	0	0	174
Epsilon	0	0	0	0	0	2	0	3	5
Eta	0	0	0	0	0	0	0	1	1
Lambda	0	0	0	0	1	0	0	0	1
Omicron	0	0	0	0	0	341 227	0	597	341 824
Zeta	0	0	0	0	0	0	0	1	1
Delta-Kappa	0	3	0	0	0	0	0	0	3
Delta-Zeta	0	2	0	0	0	0	0	0	2
Alpha-Epsilon	0	0	0	0	0	10	0	0	10
Omicron-Alpha	0	0	0	0	0	25	0	0	25
Omicron-Delta	0	0	0	0	0	3	0	0	3
Omicron-Epsilon	0	0	0	0	0	609	0	0	609
Omicron-Zeta	0	0	0	0	0	10	0	0	10
UP	0	4	0	0	0	1699	0	524	2227
Total	2	180	0	0	1	343 592	0	1126	344 901

Abbreviations: GISAID, Global Initiative on Sharing Avian Influenza Data; HAI, haplotype-based artificial intelligence; UA, unassigned variant; UP, unidentifiable variant.

^a^ Conducted in the prospective data set with 344 901 viruses collected after March 14, 2022, and downloaded on May 18, 2022. Concordances of HAI identifications prior to postidentification modification with GISAID assignments are shown in eTable 5 in Supplement 1.

^b^ Posterial probability threshold = 0.99.

### Observed of Recombinant Types

Co-infection could lead to the recombination of 2 variants and the formation of a recombinant, which could empirically be observed as an MV. To identify specific mixtures, we defined a specific MV if the virus carried at least 1 mutating polymutant unique to respective variants. The application of postidentification processing identified a set of potential recombinants (Table 3). The most frequently occurring recombinant type among all 657 MVs was Omicron-Epsilon (609 recombinants [92.7%]). Among all recombinants, the likely most well-known and controversial recombinant is the Omicron-Delta recombinant.^29,30,31^ Profiling Delta and Omicron polymutants on these 2 recombinants (eTable 19 in Supplement 1), we found that the virus carried L452R and I82T polymutants unique to Delta, while the remaining polymutants were unique to Omicron. Similarly, the Omicron-Alpha recombinants carried T183I, S982A, R52I, D3L, and S235F mutations unique to Alpha, while Omicron-Zeta recombinants carried L71F, A119S, and M234I mutations unique to Zeta (eTable 20 in Supplement 1). Omicron-Epsilon recombinants carried T85I, I65V, L452R, R57H, and T205I mutations unique to Epsilon (eTable 21 in Supplement 1). To profile the epidemiological distribution of Omicron-Epsilon recombinants, we tabulated their geographic and temporal distribution with respect to collection date and location (eTable 22 in Supplement 1).

Crosstabulating MVs with assigned lineages (eTable 23 in Supplement 1), we noted that Delta recombinants with Kappa and Zeta variants were more frequently assigned to AY lineages and Omicron recombinants were more frequently assigned BA lineages. An Omicron-Epsilon recombinant was assigned to BA.4, while an Omicron-Alpha recombinant was assigned to BA.5.

### Acquisition of New Mutations

**Figure 1.**
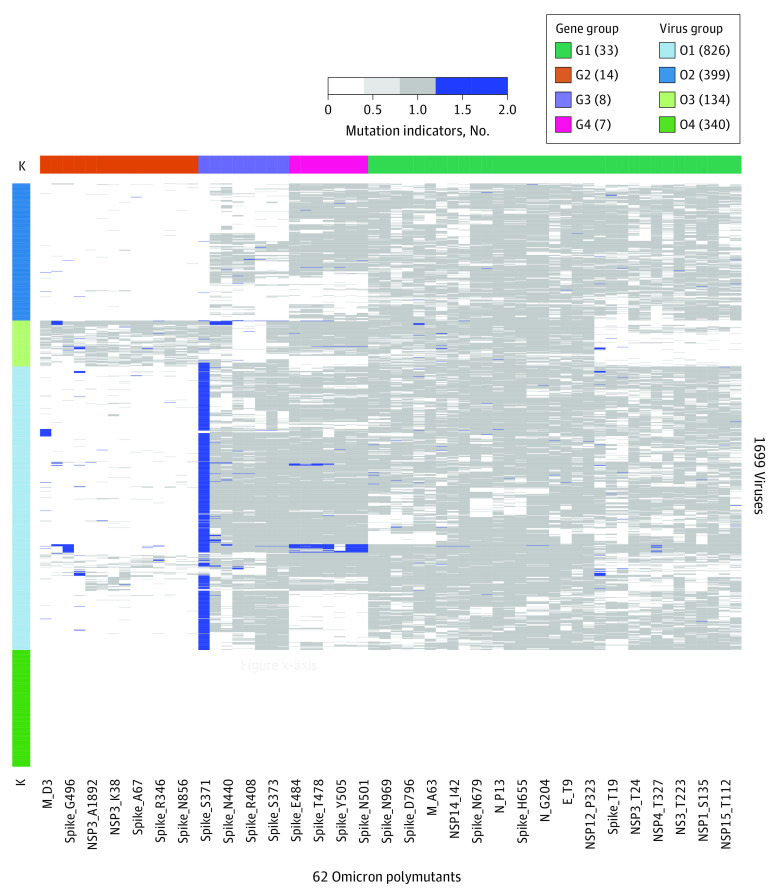
Newly Acquired Mutations New mutations were found among a group of 1699 Omicron viruses that had evolving core haplotypes and rendered the haplotype-based artificial intelligence model unable to identify the variant. In the heat map, wild types, Omicron mutations, and new mutations are coded as 0, 1, and 2 and are colored as white, gray, and blue, respectively.

Among 343 592 Omicron viruses, 1699 viruses were found to be unidentifiable by HAI because the observed haplotypes were not part of any previously observed Omicron core haplotype. Thus, we hypothesized that some Omicron viruses may have rapidly acquired new mutations. To identify new mutations acquired by these Omicron viruses, we applied an unsupervised learning technique to organize a matrix of mutation indicators for amino acids in reference virus, Omicron-specific mutations, and newly acquired mutations (Figure 1). Biclustering of polymutant similarities was associated with clustered viruses (O1, O2, O3, and O4) and clustered Omicron polymutants (G1, G2, G3, and G4). Other than viruses in cluster O4, most viruses displayed sporadic mutations; however, S371 in the spike protein acquired a new mutation, S371F, while most Omicron viruses exhibited S371L mutations, in addition to a few random substitutions (Y, A, C, and deletion). E484, S477, T478, Q493, Y505, Q498, and N501 also acquired comparatively few such mutations. To gain insights into the mutation at S371, we crosstabulated collection dates and countries and found that this mutation was first sequenced in Europe and was spreading to other countries. Viruses in the group O4 were assigned to lineages and Omicron, but no polymutants were listed, which may be associated with data processing errors at GISAID.

In crosstabulating cluster group (O1, O2, O3, and O4) with lineages (eTable 24 in Supplement 1), we found that most viruses in the O1 group belonged to BA.1 and BA.2, but the group also included 1 BA.4 and 3 BA.5 variants, in addition to including the 8 XE variant. The viruses in group O2 were predominantly BA.1 variants, while those in group O3 were predominantly BA.2 variants.

### Expanding Novel Mutations

**Figure 2.**
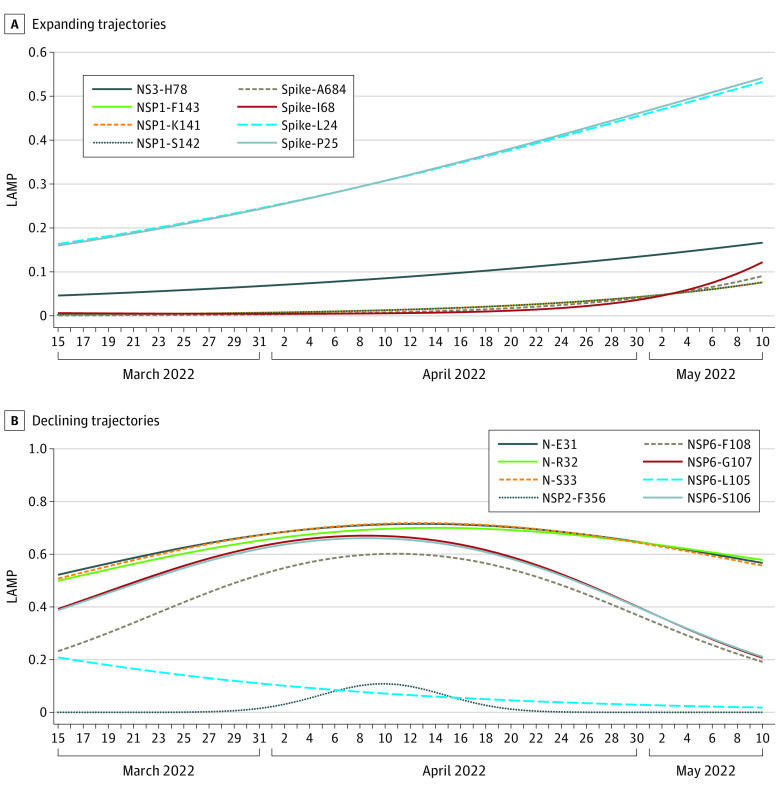
Expanding and Declining Polymutants Among 524 unassigned and unidentifiable viruses, 16 ab initio mutations were found; 8 were expanding, while the rest had negative trajectories. LAMP indicates locally averaged mutation percentage.

Among all 1126 unassigned viruses, 524 viruses were deemed unidentifiable by the HAI model. These unassigned and unidentifiable viruses may have acquired novel mutations. Applying SLS, we modeled the temporal expansions of polymutants in this set and selected 56 polymutants by their significant and substantial temporal expansions (*P* value < .05 and LAMP_max _> 0.5). Excluding polymutants that were part of variant-specific core polymutants, we found 16 new polymutants (N-E31, N-R32, N-S33, NS3-H78, NSP1-F143, NSP1-K141, NSP1-S142, NSP2-F356, NSP6-F108. NSP6-G107, NSP6-L105, NSP6-S106, spike-A684, spike-I68, spike-L24, and spike-P25) (eTable 25 in Supplement 1). Application of unsupervised learning yielded 6 groups of polymutants by their temporal trends (eFigure 3 in Supplement 1). Visually, 8 polymutants (NSP1-K141/S142/F143, NS3-H78, and spike-L24/P25/I68/A684) in groups 1, 3, and 4 were expanding (Figure 2), while the remaining polymutants, with varying LAMP levels (NSP2-F356, NSP6-L105/S106/G107/F108, and N-E31/R32/S33) were declining (Figure 2). L24 and P25 of the spike protein were expanding at faster trajectories, while H78 of NS3 was expanding rapidly. There were 2 spike polymutants (I68 and A684) and 3 polymutants (K141, S142, F143) that overlapped with NSP1 that were increasing. The remaining 8 polymutants, with varying LAMP levels, were declining.

## Discussion

In this cross-sectional study, we described an HAI model for identifying novel SARS-COV-2 variants that was trained and validated with approximately 10 million viral sequences. Applying HAI to a prospective set of viruses collected between March 15 and May 18, 2022, we found that the HAI model achieved 93% concordance with GISAID assignments, with a 0.003% discordance rate. The HAI model was able to identify MVs and variants with novel mutations. From more than 340 000 viruses, the HAI model identified 7 unique MVs (Omicron-Alpha, Omicron-Delta, Omicron-Epsilon, Omicron-Zeta, Alpha-Epsilon, Delta-Kappa, and Delta-Zeta). It was also of interest to discover that Omicron polymutants continued to acquire novel mutations. For example, S371 in the spike protein was commonly substituted with S371L among Omicron viruses but was subsequently increasingly substituted by S371F. These S371L/F mutations, commonly observed for BA.1 and BA.2, may have been associated with a perturbation of spike trimer conformational dynamics.^32^ Additionally, 8 novel mutations (NSP1-K141/S142/F143, NS3-H78, and spike-L24/P25/I68/A684) appeared to be increasing in prevalence recently and may require careful monitoring.

HAI treated GISAID assignment as a standard criterion in the training process, although some assignments may be subject to misclassification errors. Fortunately, such misassignments may be few in the current GISAID given that co-infections were exceptionally rare until recent months. Hence, imperfect training data may have had limited impact on the validity of the HAI. Furthermore, its empirical nature, relying on statistical learning strategies, tends to be robust despite a few misclassification errors.

The HAI method may be routinely used to identify important MVs in the future. For example, the Delta variant carries mutations that are associated with disease severity and hospitalization risk.^33^ While Delta-Omicron recombinants are rare thus far, a highly transmissible variant, like Omicron, if recombining with virulent variants,^33^ would be cause for concern. Hence, early identification of such MVs may be crucial for effective public health planning.

The approached described in this study is complementary to phylogenic-based variant assignment by GISAID, with the added benefit of timely identification of novel variants that may not otherwise become apparent at early stages. Rapid identification of these variants via the HAI, in addition to geographic and temporal localization, may facilitate correlation of specific variants with clinical outcomes assessable through electronic health records.^24,34^ It has the potential to inform a broad range of public health strategies, including heightened surveillance, diagnostics, therapeutics, and even vaccine strategies depending on the variant haplotype.

### Limitations

While the HAI model demonstrated clear advantages, we need to be mindful of this study’s limitations. Perhaps the most substantial limitation was that an identified MV may not necessarily arise from recombination due to co-infection. An alternative process is that reinfection may lead to an MV. Sequence contamination may falsify an MV, but such MVs may be rare (1 or 2 copies). Hence, identified MVs may need to be investigated experimentally. Another limitation is that the current HAI is trained and validated with global data collected over the past 2 years. Its identification performance may need to be optimized for specific geographic regions, and it may need to be updated continuously to incorporate newly collected viral sequences. For example, since May 18, 2022, Omicron has evolved into multiple lineages, and HAI may need to account for these lineages. Additionally, our HAI model has several tunable parameters, which may be associated with identification performance. Further research may be necessary to improve robustness and performance of HAI identifications.

## Conclusions

In this cross-sectional study, we described an HAI model to detect novel SARS-COV-2 variants. Applying HAI to 344 901 sequences submitted to GISAID globally from March 15 to May 18, 2022, we found that several new MVs were circulating globally and that several novel mutations were expanding recently. We have implemented the HAI model in a web-based calculator^35^ for use by the community to facilitate discovery of novel variants.

## Notes

Supplement 1.
eMethods.
**eTable 1.** A List of Known Variants Assigned by GISAID **eTable 2.** Haplotype Frequencies Among Alpha Viruses **eTable 3.** Haplotype Frequencies Among Beta Viruses **eTable 4.** Haplotype Frequencies Among Delta Viruses **eTable 5.** Haplotype Frequencies Among Epsilon Viruses **eTable 6.** Haplotype Frequencies Among Eta Viruses **eTable 7.** Haplotype Frequencies Among Gamma Viruses **eTable 8.** Haplotype Frequencies Among GH/490R Viruses **eTable 9.** Haplotype Frequencies Among Iota Viruses **eTable 10.** Haplotype Frequencies Among Kappa Viruses **eTable 11.** Haplotype Frequencies Among Lambda Viruses **eTable 12.** Haplotype Frequencies Among Mu Viruses **eTable 13.** Haplotype Frequencies Among Omicron Viruses **eTable 14.** Haplotype Frequencies Among Theta Viruses **eTable 15.** Haplotype Frequencies Among Zeta Viruses **eTable 16.** Concordance Analysis in the Training Set **eTable 17.** Concordance Analysis in the Validation Set **eTable 18.** Concordance Analysis in the Prospective Set **eTable 19.** Four Mixture Variants with Omicron-Delta **eTable 20.** Four Mixture Variants with Omicron-Alpha **eTable 21.** Four Mixture Variants with Omicron-Zeta **eTable 22.** Four Mixture Variants with Omicron-Epsilon **eTable 23.** Mixture Variant and Corresponding Lineages **eTable 24.** Unidentifiable Omicron Viruses and Corresponding Lineages **eTable 25.** New Mutations Among Variant-Unassigned and Unidentifiable Viruses **eFigure 1.** Heatmap-Representation of Selected Polymutant Temporal Profile From January 1, 2020 to March 14, 2022 Within Every Variant **eFigure 2.** Misclassification Errors by Haplotype-Based Variant Prediction (HVP), When the Prediction Probability Threshold Value is Set at 0.9 to 1 **eFigure 3.** Temporal Patterns of Sixteen Polymutants Identified From Variant-Unassigned 524 Viruses That Are Unpredictable by HAI, Excluding Those Core Polymutants of All Fourteen Variants

Supplement 2.
Data Sharing Statement
