# Supplementary material for: Using Haplotype-Based Artificial Intelligence to Evaluate SARS-CoV-2 Novel Variants and Mutations
Source: JAMA Netw Open. 2023 Feb 21;6(2):e230191. doi: 10.1001/jamanetworkopen.2023.0191 (PMC9945077; doi:10.1001/jamanetworkopen.2023.0191)
Supplement: Supplement 1. — eMethods. eTable 1. A List of Known Variants Assigned by GISAID eTable 2. Haplotype Frequencies Among Alpha Viruses eTable 3. Haplotype Frequencies Among Beta Viruses eTable 4. Haplotype Frequencies Among Delta Viruses eTable 5. Haplotype Frequencies Among Epsilon Viruses eTable 6. Haplotype Frequencies Among Eta Viruses eTable 7. Haplotype Frequencies Among Gamma Viruses eTable 8. Haplotype Frequencies Among GH/490R Viruses eTable 9. Haplotype Frequencies Among Iota Viruses eTable 10. Haplotype Frequencies Among Kappa Viruses eTable 11. Haplotype Frequencies Among Lambda Viruses eTable 12. Haplotype Frequencies Among Mu Viruses eTable 13. Haplotype Frequencies Among Omicron Viruses eTable 14. Haplotype Frequencies Among Theta Viruses eTable 15. Haplotype Frequencies Among Zeta Viruses eTable 16. Concordance Analysis in the Training Set eTable 17. Concordance Analysis in the Validation Set eTable 18. Concordance Analysis in the Prospective Set eTable 19. Four Mixture Variants with Omicron-Delta eTable 20. Four Mixture Variants with Omicron-Alpha eTable 21. Four Mixture Variants with Omicron-Zeta eTable 22. Four Mixture Variants with Omicron-Epsilon eTable 23. Mixture Variant and Corresponding Lineages eTable 24. Unidentifiable Omicron Viruses and Corresponding Lineages eTable 25. New Mutations Among Variant-Unassigned and Unidentifiable Viruses eFigure 1. Heatmap-Representation of Selected Polymutant Temporal Profile From January 1, 2020 to March 14, 2022 Within Every Variant eFigure 2. Misclassification Errors by Haplotype-Based Variant Prediction (HVP), When the Prediction Probability Threshold Value is Set at 0.9 to 1 eFigure 3. Temporal Patterns of Sixteen Polymutants Identified From Variant-Unassigned 524 Viruses That Are Unpredictable by HAI, Excluding Those Core Polymutants of All Fourteen Variants [file jamanetwopen-e230191-s001.pdf]

Supplemental Online Content

Zhao LP, Cohen S, Zhao M, et al. Using haplotype-based artificial intelligence to evaluate SARS-CoV-2 novel variants and mutations. *JAMA Netw Open*. 2023;6(2):e230191. doi:10.1001/jamanetworkopen.2023.0191

eMethods.

eTable 1. A List of Known Variants Assigned by GISAID

eTable 2. Haplotype Frequencies Among Alpha Viruses

eTable 3. Haplotype Frequencies Among Beta Viruses

eTable 4. Haplotype Frequencies Among Delta Viruses

eTable 5. Haplotype Frequencies Among Epsilon Viruses

eTable 6. Haplotype Frequencies Among Eta Viruses

eTable 7. Haplotype Frequencies Among Gamma Viruses

eTable 8. Haplotype Frequencies Among GH/490R Viruses

eTable 9. Haplotype Frequencies Among Iota Viruses

eTable 10. Haplotype Frequencies Among Kappa Viruses

eTable 11. Haplotype Frequencies Among Lambda Viruses

eTable 12. Haplotype Frequencies Among Mu Viruses

eTable 13. Haplotype Frequencies Among Omicron Viruses

eTable 14. Haplotype Frequencies Among Theta Viruses

eTable 15. Haplotype Frequencies Among Zeta Viruses

eTable 16. Concordance Analysis in the Training Set

eTable 17. Concordance Analysis in the Validation Set

eTable 18. Concordance Analysis in the Prospective Set

eTable 19. Four Mixture Variants with Omicron-Delta

eTable 20. Four Mixture Variants with Omicron-Alpha

eTable 21. Four Mixture Variants with Omicron-Zeta

eTable 22. Four Mixture Variants with Omicron-Epsilon

eTable 23. Mixture Variant and Corresponding Lineages

eTable 24. Unidentifiable Omicron Viruses and Corresponding Lineages

eTable 25. New Mutations Among Variant-Unassigned and Unidentifiable Viruses

eFigure 1. Heatmap-Representation of Selected Polymutant Temporal Profile From January 1, 2020 to March 14, 2022 Within Every Variant

© 2023 Zhao LP et al. *JAMA Network Open.*

eFigure 2. Misclassification Errors by Haplotype-Based Variant Prediction (HVP), When the Prediction Probability Threshold Value is Set at 0.9 to 1

eFigure 3. Temporal Patterns of Sixteen Polymutants Identified From Variant-Unassigned 524 Viruses That Are Unpredictable by HAI, Excluding Those Core Polymutants of All Fourteen Variants

This supplemental material has been provided by the authors to give readers additional information about their work.

© 2023 Zhao LP et al. *JAMA Network Open.*

eMethods

Polymutants. GISAID is continuously receiving and archiving viral sequences globally, along with sample-specific metadata. Besides date and location of collecting viruses, the pertinent data to this project are extracted substituting amino acids (known as "AA.substitutions"), from aligning nucleotide sequence and translating nucleotides to amino acids by GISAID. Upon obtaining the text string on all substituting amino acids, we process all individual samples, and align all substitutions in a matrix form. In comparison with amino acids in the reference sequence, we convert the amino acid substitution matrix into a matrix of binary indicators (0, 1) for wildtype and mutant type, respectively. Note that most amino acids are monomorphic, some take one mutant type, and very few amino acids may have more than two substituting mutants. We call an amino acid a polymutant if it includes three or more mutating amino acids in the study population. While being conceptually straightforward, converting text strings of AA.substitutions into polymutant matrix and a matrix of binary polymutant indicators enables computations required by Statistical Learning Strategy (SLS).

Modeling Temporal Trends. Each polymutant has a specific temporal expansion pattern. Let binary indicator $y_{jt}(=1 \text{ or } 0)$ denote the presence or absence the of mutant type, respectively, observed at time *t* for the *j*th polymutant. To model the non-linear temporal expansion, we applied a generalized additive model (GAM) to regress the mutant indicator over sample collection time via the following probability model,

$$\Pr(y_{jt}=1 \mid t) = \frac{1}{1+\exp[-\alpha - s_j(t)]}, \qquad (1)$$

where α is a constant coefficient and $s_j(t)$ is a non-linear function of time *t*, and both are estimated by the restricted maximum likelihood method[1]. Upon completing the estimation, the above function is used with the estimated coefficient and non-linear function to compute the probability of observing mutating amino acid at the time *t*, yielding a *p*-value that measures if the function $s_j(t)$ deviates from zero. Also produced is the maximum proportion as $P_{\max} = \max[\Pr(y_{jt}=1 \mid t)]$. The function "gam" was used to fit the GAM (R packages MGCV [2]). The smoothing parameter $k = 7$ was chosen.

Upon fitting the GAM, SLS can use the fitted values to compute locally averaged mutation percentage (LAMP) daily from the first to the last reporting day. The temporal pattern of LAMP can be used to describe the temporal expansion of the *j*th polymutant. By using *p*-value, SLS calls a polymutant with a significant trend, if p-value < 0.05, and with a substantial presence if the maximum proportion (Pmax) is greater than 10%.

Haplotype Analysis. SARS-COV-2 is an RNA virus, i.e., a single strand, and thus multiple polymutants from the same virus share the same haplotype. Once two or more polymutants are identified, SLS converts polymutant matrix to a vector of polymutant haplotypes and computes their haplotype frequencies as part of the haplotype analysis. When confining the analysis to viruses of a specific variant, SLS can generate variant specific haplotype frequencies, denoted as $f(h \mid \text{variant})$.

Bayes' Prediction Probability. When predicting variant type based on viral sequences or polymutant haplotype (*H*), the haplotype-based artificial intelligence (HAI) relies on the following posterior probability:

$$\Pr(V \mid H=h) = \frac{f(H=h \mid \text{Variant}=V)\, p(\text{Variant}=V)}{\sum_V f(H=h \mid \text{Variant}=V)\, p(\text{Variant}=V)}, \qquad (2)$$

in which the summation is over all possible variants, $f(H=h \mid \text{Variant}=V)$ is an empirically estimated haplotype frequency of a specific viral variant, and $p(\text{Variant}=V)$ is the proportion of the variant. Note that HAI treats variant-unassigned viruses as a separate class. On each virus, HAI computes an array of variant-specific prediction probabilities. If a prediction probability exceeds 0.99,

© 2023 Zhao LP et al. *JAMA Network Open.*

HAI predicts the corresponding variant. If the prediction probability for the variant-unassigned viruses exceeds 0.99, HAI predicts that the corresponding viruses do not have any known variant assignment, i.e., unpredictable (**UP**). Otherwise, HAI predicts that viruses include a mixture variant (**MV**) of two or more variant-specific haplotypes. Note that the posterior probability above (2) is strictly derived under the assumption that all variants are exclusive of each other, but, when dealing with recombinants, the quantity on the right hand side (2) is preferably interpreted as a risk score, in which the summation in the denominator serves as a normalizing factor so that the summation of all risk scores equals one.

Post-prediction modification. Among those MV predictions, their polymutant haplotypes include polymutants of two or more variants, some of which are recombinants. To tease out which MVs are recombinants, HAI utilizes a post-prediction modification. It extracts variant-specific polymutants. We call a MV recombinant if the polymutant includes mutating amino acids from two variants. For all other MVs, they will be re-assigned to be of specific variants.

Statistical Software. The statistical package R (version: R 4.2.1) and RStudio (Release 782775e, 2022-07-22) are used to implement all computational procedures in the HAI.

© 2023 Zhao LP et al. *JAMA Network Open.*

eTable 1. A List of Known Variants Assigned by GISAID

Fourteen variants established at GISAID and their basic annotations: WHO nomeclaure, clade/lineages assigned by phylogenic analysis, location(s) where the variant is first reported, and current designation by CDC

ID	WHO	Clade/Lineages	First Detect in	Variant
1	Alpha	B.1.1.7+Q.*	United Kingdom	VOC
2	Beta	GH/501Y.V2 (B.1.351+B.1.351.2+B.1.351.3)	South Africa	VOC
3	Delta	GK (B.1.617.2+AY.*)	India	VOC
4	Epsilon	GH/452R.V1 (B.1.429+B.1.427)	USA/California	VOI
5	Eta	G/484K.V3 (B.1.525)	UK/Nigeria	VOI
6	Gamma	GR/501Y.V3 (P.1+P.1.*)	Brazil/Japan	VOC
7	GH/490R	(B.1.640+B.1.640.*)	Congo/France	VUM
8	Iota	GH/253G.V1 (B.1.526)	USA/New York	VOI
9	Kappa	G/452R.V3 (B.1.617.1)	India	VOI
10	Lambda	GR/452Q.V1 (C.37+C.37.1)	Peru	VOI
11	Mu	GH (B.1.621+B.1.621.1)	Colombia	VOI
12	Omicron	GRA (B.1.1.529+BA.*)	Botswana/South Africa/Hong Kong	VOC
13	Theta	GR/1092K.V1 (P.3)	Philippines	VOI
14	Zeta	GR/484K.V2 (P.2)	Brazil	VOI

© 2023 Zhao LP et al. *JAMA Network Open.*

eTable 2. Haplotype Frequencies Among Alpha Viruses

alpha. Haplotype frequencies of core haplotypes associated with alpha-specific polymutants, for those haplotypes with 0.1% haplotype frequency.

ID	Count	Freq	Mut	NSP3 T183	NSP3 A890	NSP3 I1412	NSP12 P227	NSP12 P323	NSP13 K460	Spike N501	Spike A570	Spike D614	Spike P681	Spike T716	Spike S982	Spike D1118	NS8 R52	NS8 Y73	N D3	N R203	N G204	N S235
1	293153	51.06%	17	**I**	**D**	**T**	**P**	**L**	**K**	**Y**	**D**	**G**	**H**	**I**	**A**	**H**	**I**	**C**	**L**	**K**	**R**	**F**
2	105858	18.44%	18	.	.	.	.	.	R	.	.	.	.	.	.	.	.	.	.	.	.	.
3	67800	11.81%	18	.	.	.	L	.	.	.	.	.	.	.	.	.	.	.	.	.	.	.
4	36990	6.44%	17	.	.	.	.	.	.	.	.	.	.	.	.	.	.	.	.	.	P	.
5	4410	0.77%	16	.	.	I	.	.	.	.	.	.	.	.	.	.	.	.	.	.	.	.
6	4203	0.73%	16	.	.	.	.	.	.	N	.	.	.	.	.	.	.	.	.	.	.	.
7	3217	0.56%	16	.	.	.	.	.	.	.	.	.	.	.	.	.	.	.	D	.	.	.
8	2174	0.38%	15	.	.	.	.	.	.	.	.	.	.	.	.	.	.	.	.	R	G	.
9	2081	0.36%	17	.	.	I	.	.	R	.	.	.	.	.	.	.	.	.	.	.	.	.
10	1904	0.33%	17	.	.	.	.	.	R	N	.	.	.	.	.	.	.	.	.	.	.	.
11	1777	0.31%	17	.	.	.	L	.	.	N	.	.	.	.	.	.	.	.	.	.	.	.
12	1524	0.27%	17	.	.	I	L	.	.	.	.	.	.	.	.	.	.	.	.	.	.	.
13	1361	0.24%	17	.	.	.	.	.	.	.	.	.	.	.	.	.	.	.	.	E	P	.
14	1226	0.21%	16	.	.	.	.	.	.	.	.	.	.	.	.	.	R	.	.	.	.	.
15	1086	0.19%	16	.	.	.	.	P	.	.	.	.	.	.	.	.	.	.	.	.	.	.
16	1013	0.18%	14	.	.	.	.	.	.	.	.	.	.	.	.	.	.	.	.	R	G	S
17	947	0.16%	17	.	.	.	L	.	.	.	.	.	.	.	.	.	.	.	D	.	.	.
18	942	0.16%	17	.	.	.	.	.	R	.	.	.	.	.	.	.	.	.	D	.	.	.
19	847	0.15%	16	.	.	.	L	.	.	.	.	.	.	T	S	.	.	.	.	.	.	.
20	810	0.14%	17	.	.	.	.	.	.	.	.	.	.	.	.	Y	.	.	.	.	.	.
21	786	0.14%	18	.	.	.	.	.	R	.	.	.	R	.	.	.	.	.	.	.	.	.
22	719	0.13%	16	.	.	.	.	.	R	.	.	.	.	.	.	.	R	Y	.	.	.	.
23	710	0.12%	15	.	.	.	.	.	.	.	.	.	.	.	.	.	R	Y	.	.	.	.
24	677	0.12%	16	.	A	.	.	.	.	.	.	.	.	.	.	.	.	.	.	.	.	.
25	677	0.12%	16	.	.	.	.	.	.	.	.	.	.	.	.	.	.	.	.	.	.	S
26	647	0.11%	16	T	.	.	.	.	.	.	.	.	.	.	.	.	.	.	.	.	.	.
27	631	0.11%	18	.	.	.	L	.	.	.	.	.	.	.	.	Y	.	.	.	.	.	.
28	617	0.11%	15	.	.	.	.	.	.	.	.	.	.	T	S	.	.	.	.	.	.	.
29	592	0.10%	14	.	.	.	.	.	.	.	.	.	.	.	.	.	.	.	D	R	G	.
30	587	0.10%	16	.	.	.	.	.	R	.	.	.	.	T	S	.	.	.	.	.	.	.

© 2023 Zhao LP et al. *JAMA Network Open.*

eTable 3. Haplotype Frequencies Among Beta Viruses

Beta. Haplotype frequencies of core haplotypes associated with variant-specific polymutants, for those haplotypes with 0.1% haplotype frequency.

ID	**Count**	**Freq**	**Mut**	NSP2 T85	NSP3 S794	NSP3 K837	NSP3 N1778	NSP5 K90	NSP12 P323	NSP13 T588	Spike L18	Spike D80	Spike D215	Spike K417	Spike E484	Spike N501	Spike D614	Spike A701	NS3 Q57	NS3 S171	E P71	NS8 I121	N T205
1	3323	15.78%	15	**I**	**S**	**N**	**N**	**R**	**L**	**T**	**L**	**A**	**G**	**N**	**K**	**Y**	**G**	**V**	**H**	**L**	**L**	**I**	**I**
2	2770	13.16%	16	.	.	.	.	.	.	.	F	.	.	.	.	.	.	.	.	.	.	.	.
3	1733	8.23%	16	.	L	.	.	.	.	.	.	.	.	.	.	.	.	.	.	.	.	.	.
4	1703	8.09%	17	.	.	.	S	.	.	.	F	.	.	.	.	.	.	.	.	.	.	.	.
5	1411	6.70%	17	.	.	.	.	.	.	I	.	.	.	.	.	.	.	.	.	.	.	L	.
6	982	4.66%	17	.	.	.	.	.	.	.	F	.	.	.	.	.	.	.	.	.	.	L	.
7	933	4.43%	16	.	.	.	.	.	H	.	F	.	.	.	.	.	.	.	.	.	.	.	.
8	385	1.83%	13	.	.	.	.	.	.	.	.	.	.	.	E	N	.	.	.	.	.	.	.
9	372	1.77%	16	.	.	.	.	.	.	.	.	.	.	.	.	.	.	.	.	.	.	L	.
10	276	1.31%	18	.	.	.	S	.	.	.	F	.	.	.	.	.	.	.	.	.	.	L	.
11	270	1.28%	16	.	.	.	.	.	.	I	.	.	.	.	.	.	.	.	.	.	.	.	.
12	241	1.14%	14	.	.	.	.	.	.	.	F	.	.	.	E	N	.	.	.	.	.	.	.
13	220	1.04%	13	.	.	.	.	.	.	.	.	D	D	.	.	.	.	.	.	.	.	.	.
14	209	0.99%	17	.	L	.	.	.	.	.	.	.	.	.	.	.	.	.	.	.	.	L	.
15	195	0.93%	14	.	.	K	.	.	.	I	.	.	.	K	.	.	.	.	.	S	.	L	.
16	187	0.89%	15	.	.	.	.	.	.	I	.	.	.	K	.	.	.	.	.	S	.	L	.
17	118	0.56%	15	.	.	.	S	.	.	.	F	.	.	.	E	N	.	.	.	.	.	.	.
18	115	0.55%	14	.	.	.	.	.	.	.	.	.	D	.	.	.	.	.	.	.	.	.	.
19	106	0.50%	14	.	L	.	.	.	.	.	.	.	.	.	E	N	.	.	.	.	.	.	.
20	98	0.47%	15	.	.	.	.	.	.	I	.	.	.	.	E	N	.	.	.	.	.	L	.
21	96	0.46%	14	.	.	.	.	.	.	.	.	.	.	.	.	.	.	.	.	S	.	.	.
22	95	0.45%	15	.	.	.	.	.	.	.	F	.	.	.	.	.	.	.	.	S	.	.	.
23	93	0.44%	17	.	.	.	.	.	H	.	F	.	.	.	.	.	.	.	.	.	.	L	.
24	83	0.39%	14	.	.	.	.	.	.	.	.	.	.	.	E	.	.	.	.	.	.	.	.
25	77	0.37%	14	.	.	.	.	.	.	.	.	.	.	K	.	.	.	.	.	.	.	.	.
26	72	0.34%	14	.	.	.	.	.	H	.	F	.	.	.	E	N	.	.	.	.	.	.	.
27	71	0.34%	14	.	.	.	.	.	.	.	.	.	.	.	.	.	.	.	.	.	.	.	T
28	70	0.33%	15	.	.	.	.	.	.	.	F	.	.	K	.	.	.	.	.	.	.	.	.
29	65	0.31%	10	.	.	K	.	.	.	.	.	D	D	.	.	.	.	A	.	S	.	.	.
30	61	0.29%	15	.	.	K	.	.	.	.	F	.	.	.	.	.	.	.	.	.	.	.	.
31	60	0.28%	12	.	.	K	.	.	.	.	.	.	.	.	E	N	.	.	.	.	.	.	.
32	56	0.27%	15	.	.	.	.	.	.	.	F	.	.	.	E	N	.	.	.	.	.	L	.
33	54	0.26%	14	.	.	.	.	.	.	.	.	.	.	.	E	N	.	.	.	.	.	L	.
34	54	0.26%	10	.	.	.	.	.	.	.	.	D	D	K	E	N	.	.	.	.	.	.	.
35	52	0.25%	14	.	.	K	.	.	.	.	.	.	.	.	.	.	.	.	.	.	.	.	.
36	49	0.23%	14	.	.	.	.	.	.	.	.	.	.	.	.	.	.	.	Q	.	.	.	.
37	48	0.23%	14	.	.	K	.	.	.	I	.	.	.	.	E	N	.	.	.	.	.	L	.
38	42	0.20%	16	.	.	K	.	.	.	I	.	.	.	.	.	.	.	.	.	.	.	L	.
39	42	0.20%	12	.	.	.	.	.	.	.	.	D	D	.	.	.	.	.	.	.	P	.	.

© 2023 Zhao LP et al. *JAMA Network Open.*

eTable 4. Haplotype Frequencies Among Delta Viruses

Delta. Haplotype frequencies of core haplotypes associated with variant-specific polymutants, for those haplotypes with 0.1% haplotype frequency.

ID	Count	Freq	Mut	NSP3 A488	NSP3 P822	NSP3 P1228	NSP3 P1469	NSP3 A1711	NSP4 V167	NSP4 A446	NSP4 T492	NSP6 T77	NSP6 V149	NSP12 P323	NSP12 G671	NSP13 P77	NSP14 A394	Spike T19	Spike T95	Spike G142	Spike E156	Spike A222	Spike L452	Spike T478	Spike D614	Spike P681	Spike D950	NS3 S26	M I82	NS7a V82	NS7a T120	NS7b T40	N D63	N R203	N G215	N D377
1	482881	22.55%	27	S	P	L	S	A	L	A	I	A	V	L	S	L	V	R	T	D	G	A	R	K	G	R	N	L	T	A	I	I	G	M	C	Y
2	276071	12.89%	29	.	.	.	.	V	.	.	.	.	.	.	.	.	.	.	I	.	.	.	.	.	.	.	.	.	.	.	.	.	.	.	.	.
3	233724	10.92%	26	.	.	.	.	.	.	.	.	.	.	.	.	.	.	.	.	G	.	.	.	.	.	.	.	.	.	.	.	.	.	.	.	.
4	205786	9.61%	28	.	.	.	.	.	.	.	.	.	.	.	.	.	.	.	I	.	.	.	.	.	.	.	.	.	.	.	.	.	.	.	.	.
5	74239	3.47%	22	A	L	P	P	.	V	V	T	T	A	.	.	.	A	.	.	.	.	V	.	.	.	.	.	.	.	.	.	T	.	.	G	.
6	64840	3.03%	27	.	.	.	.	.	.	.	.	.	.	.	.	.	.	.	I	G	.	.	.	.	.	.	.	.	.	.	.	.	.	.	.	.
7	50506	2.36%	30	.	.	.	.	V	.	.	.	.	.	.	.	.	.	.	I	.	.	V	.	.	.	.	.	.	.	.	.	.	.	.	.	.
8	37778	1.76%	28	.	.	.	.	V	.	.	.	.	.	.	.	.	.	.	I	G	.	.	.	.	.	.	.	.	.	.	.	.	.	.	.	.
9	37472	1.75%	27	.	.	.	.	V	.	.	.	.	.	.	.	.	.	.	.	G	.	.	.	.	.	.	.	.	.	.	.	.	.	.	.	.
10	33301	1.56%	21	A	L	P	P	.	V	V	T	T	A	.	.	.	A	.	.	G	.	V	.	.	.	.	.	.	.	.	.	T	.	.	G	.
11	30783	1.44%	25	.	.	.	.	.	.	.	.	.	.	.	.	.	.	.	.	G	E	.	.	.	.	.	.	.	.	.	.	.	.	.	.	.
12	15479	0.72%	21	A	L	P	P	.	V	V	T	T	A	.	.	.	A	.	.	.	.	.	.	.	.	.	.	.	.	.	.	T	.	.	G	.
13	11681	0.55%	26	.	.	P	.	.	.	.	.	.	.	.	.	.	.	.	.	.	.	.	.	.	.	.	.	.	.	.	.	.	.	.	.	.
14	10633	0.50%	25	.	.	.	.	.	.	.	.	.	.	.	.	.	.	.	.	G	.	.	.	.	.	.	D	.	.	.	.	.	.	.	.	.
15	10548	0.49%	20	A	L	P	P	.	V	V	T	T	A	.	.	.	A	.	.	G	.	.	.	.	.	.	.	.	.	.	.	T	.	.	G	.
16	10494	0.49%	26	.	.	.	.	.	.	.	.	.	.	.	.	.	.	.	.	.	E	.	.	.	.	.	.	.	.	.	.	.	.	.	.	.
17	8398	0.39%	24	.	.	.	.	.	.	.	.	.	.	.	.	.	.	.	.	G	.	.	.	.	.	.	.	.	.	V	T	.	.	.	.	.
18	7751	0.36%	24	.	.	.	.	.	.	.	.	.	.	.	.	.	.	.	.	G	.	.	.	.	.	.	D	.	.	.	.	.	D	.	.	.
19	6618	0.31%	28	.	.	.	.	V	.	.	.	.	.	.	G	.	.	.	I	.	.	.	.	.	.	.	.	.	.	.	.	.	.	.	.	.
20	6330	0.30%	25	.	.	.	.	.	.	.	.	.	.	.	.	.	.	.	.	.	.	.	.	.	.	.	.	.	.	V	T	.	.	.	.	.
21	6227	0.29%	28	.	.	.	.	.	.	.	.	.	F	.	.	.	.	.	.	.	.	.	.	.	.	.	.	.	.	.	.	.	.	.	.	.
22	6076	0.28%	27	.	.	.	.	V	.	.	.	.	.	.	.	.	.	.	I	.	.	.	.	.	.	.	.	.	.	V	T	.	.	.	.	.
23	5657	0.26%	26	.	.	.	.	V	.	.	.	.	.	.	.	.	.	.	.	G	E	.	.	.	.	.	.	.	.	.	.	.	.	.	.	.
24	5648	0.26%	23	.	.	.	.	.	.	.	.	.	.	.	.	.	.	.	.	G	E	.	.	.	.	.	.	.	.	V	T	.	.	.	.	.
25	5640	0.26%	27	.	.	.	.	.	.	.	.	.	.	.	.	.	.	.	I	.	.	.	.	.	.	.	.	.	.	V	.	.	.	.	.	.
26	5437	0.25%	27	.	.	.	.	.	.	.	.	.	.	.	.	.	.	.	I	.	E	.	.	.	.	.	.	.	.	.	.	.	.	.	.	.
27	4911	0.23%	26	.	.	.	.	.	.	.	.	.	.	.	.	.	.	.	.	.	.	.	.	.	.	.	.	.	.	V	.	.	.	.	.	.
28	4782	0.22%	28	.	.	P	.	V	.	.	.	.	.	.	.	.	.	.	I	.	.	.	.	.	.	.	.	.	.	.	.	.	.	.	.	.
29	4736	0.22%	26	.	.	.	.	.	.	.	.	.	.	.	.	.	.	.	I	G	E	.	.	.	.	.	.	.	.	.	.	.	.	.	.	.
30	4657	0.22%	26	.	.	.	P	.	.	.	.	.	.	.	.	.	.	.	.	.	.	.	.	.	.	.	.	.	.	.	.	.	.	.	.	.
31	4533	0.21%	26	.	.	.	.	.	.	.	.	.	.	.	.	.	.	.	.	.	.	.	.	.	.	.	D	.	.	.	.	.	.	.	.	.
32	4517	0.21%	25	.	.	.	.	.	.	.	.	.	.	.	.	.	.	.	.	G	.	.	.	.	.	.	.	.	.	.	.	.	D	.	.	.
33	4437	0.21%	26	.	.	.	.	.	.	.	.	.	.	.	.	.	.	.	I	G	.	.	.	.	.	.	D	.	.	.	.	.	.	.	.	.
34	4261	0.20%	28	.	.	.	.	V	.	.	.	.	.	.	.	.	.	.	I	.	E	.	.	.	.	.	.	.	.	.	.	.	.	.	.	.
35	4184	0.20%	17	A	L	P	P	.	V	.	T	T	.	.	G	P	A	.	.	.	.	.	.	.	.	.	.	.	.	.	.	T	.	.	G	.
36	4060	0.19%	27	.	.	P	.	V	.	.	.	.	.	.	G	.	.	.	I	.	.	.	.	.	.	.	.	.	.	.	.	.	.	.	.	.
37	3911	0.18%	26	.	.	.	.	V	.	.	.	.	.	.	.	.	.	.	I	.	.	.	.	.	.	.	.	.	.	V	T	T	.	.	.	.
38	3793	0.18%	19	A	L	P	P	.	V	V	T	T	A	.	.	.	A	.	.	G	E	.	.	.	.	.	.	.	.	.	.	T	.	.	G	.
39	3778	0.18%	24	.	.	.	.	.	.	.	.	.	.	.	.	.	.	.	.	G	.	.	L	T	.	.	.	.	.	.	.	.	.	.	.	.

© 2023 Zhao LP et al. *JAMA Network Open.*

eTable 5. Haplotype Frequencies Among Epsilon Viruses

Epsilon. Haplotype frequencies of core haplotypes associated with variant-specific polymutants, for those haplotypes with 0.1% haplotype frequency.

ID	Count	Freq	Mut	T85 NSP2	I65 NSP9	P323 NSP12	P53 NSP13	D260 NSP13	S13 Spike	W152 Spike	L452 Spike	D614 Spike	Q57 NS3	A65 NS8	V100 NS8	T205 N	M234 N
1	11106	30.92%	10	**I**	**V**	**L**	**P**	**Y**	**I**	**C**	**R**	**G**	**H**	**A**	**V**	**I**	**M**
2	7653	21.30%	12	.	.	.	.	.	.	.	.	.	.	.	L	.	I
3	6205	17.27%	10	.	I	.	L	.	.	.	.	.	.	.	.	.	.
4	2642	7.35%	11	.	I	.	L	.	.	.	.	.	.	V	.	.	.
5	716	1.99%	11	.	.	.	.	.	.	.	.	.	.	.	.	.	I
6	605	1.68%	9	.	.	P	.	.	.	.	.	.	.	.	.	.	.
7	497	1.38%	9	.	.	.	.	.	S	.	.	.	.	.	.	.	.
8	443	1.23%	10	.	I	.	L	.	.	W	.	.	.	V	.	.	.
9	321	0.89%	9	.	I	.	L	.	S	.	.	.	.	.	.	.	.
10	304	0.85%	11	.	.	P	.	.	.	.	.	.	.	.	L	.	I
11	272	0.76%	11	.	.	.	.	.	S	.	.	.	.	.	L	.	I
12	262	0.73%	9	.	I	.	.	.	.	.	.	.	.	.	.	.	.
13	256	0.71%	9	.	I	P	L	.	.	.	.	.	.	.	.	.	.
14	236	0.66%	9	.	.	.	.	.	.	W	.	.	.	.	.	.	.
15	169	0.47%	9	.	.	.	.	.	.	.	L	.	.	.	.	.	.
16	158	0.44%	11	.	.	.	.	.	.	W	.	.	.	.	L	.	I
17	148	0.41%	10	.	I	P	L	.	.	.	.	.	.	V	.	.	.
18	148	0.41%	8	.	I	P	.	.	.	.	.	.	.	.	.	.	.
19	141	0.39%	11	.	.	.	.	.	.	.	L	.	.	.	L	.	I
20	117	0.33%	11	.	.	.	.	.	.	.	.	.	.	V	.	.	.
21	108	0.30%	9	.	I	.	L	.	S	W	.	.	.	V	.	.	.
22	107	0.30%	9	.	.	.	.	.	.	.	.	.	.	.	.	T	.
23	98	0.27%	9	.	I	.	L	.	.	W	.	.	.	.	.	.	.
24	98	0.27%	8	.	.	.	.	.	S	W	.	.	.	.	.	.	.
25	82	0.23%	10	.	I	.	L	.	.	.	L	.	.	V	.	.	.
26	79	0.22%	11	.	I	.	.	.	.	.	.	.	.	.	L	.	I
27	78	0.22%	8	.	I	.	L	.	S	W	.	.	.	.	.	.	.
28	74	0.21%	8	.	.	.	.	D	S	.	.	.	.	.	.	.	.
29	71	0.20%	10	.	I	P	.	.	.	.	.	.	.	.	L	.	I
30	70	0.19%	10	.	I	.	L	.	S	.	.	.	.	V	.	.	.
31	64	0.18%	9	.	I	.	L	.	.	.	L	.	.	.	.	.	.
32	54	0.15%	9	.	I	.	L	.	.	W	L	.	.	V	.	.	.
33	52	0.14%	10	.	.	.	.	.	S	W	.	.	.	.	L	.	I
34	51	0.14%	9	.	.	.	.	D	.	.	.	.	.	.	.	.	.
35	46	0.13%	8	.	.	.	.	.	.	W	L	.	.	.	.	.	.
36	44	0.12%	8	.	I	.	.	.	S	.	.	.	.	.	.	.	.
37	41	0.11%	8	.	I	.	.	.	.	.	.	.	Q	.	.	.	.

© 2023 Zhao LP et al. *JAMA Network Open.*

eTable 6. Haplotype Frequencies Among Eta Viruses

Eta. Haplotype frequencies of core haplotypes associated with variant-specific polymutants, for those haplotypes with 0.1% haplotype frequency.

ID	Core Haplotypes	Coun	Freq	Mut	T1189 NSP3	K1771 NSP3	P323 NSP12	K160 NSP16	Q52 Spike	A67 Spike	E484 Spike	D614 Spike	Q677 Spike	F888 Spike	L21 E	I82 M	A12 N	I205 N
1	IKFKRVKGHLFTGI	2188	46.41%	12	I	K	F	K	R	V	K	G	H	L	F	T	G	I
2	IKFRRVKGHLFTGI	536	11.37%	13	.	.	.	R	.	.	.	.	.	.	.	.	.	.
3	IRFKRVKGHLFTGI	425	9.02%	13	.	R	.	.	.	.	.	.	.	.	.	.	.	.
4	IKFKQVKGHLLTGI	338	7.17%	10	.	.	.	.	Q	.	.	.	.	.	L	.	.	.
5	IKFKRVKGHLLTGI	204	4.33%	11	.	.	.	.	.	.	.	.	.	.	L	.	.	.
6	IKFRRVKGHLFTAT	149	3.16%	11	.	.	.	R	.	.	.	.	.	.	.	.	A	T
7	IKFKQVKGHLFTGI	90	1.91%	11	.	.	.	.	Q	.	.	.	.	.	.	.	.	.
8	IKFKRAKGHLFTGI	61	1.29%	11	.	.	.	.	.	A	.	.	.	.	.	.	.	.
9	IKFKRVKGHLFTGT	50	1.06%	11	.	.	.	.	.	.	.	.	.	.	.	.	.	T
10	IKFKRVKGHLFTAI	49	1.04%	11	.	.	.	.	.	.	.	.	.	.	.	.	A	.
11	IKFKRVEGHLFTGI	35	0.74%	11	.	.	.	.	.	.	E	.	.	.	.	.	.	.
12	IKFKQAKGHLFTGI	25	0.53%	10	.	.	.	.	Q	A	.	.	.	.	.	.	.	.
13	IKFKRVKGHLFTVI	21	0.45%	12	.	.	.	.	.	.	.	.	.	.	.	.	V	.
14	IKFKRVKGHFFTGI	20	0.42%	11	.	.	.	.	.	.	.	.	.	F	.	.	.	.
15	TKFKRVKGHLFTGI	19	0.40%	11	T	.	.	.	.	.	.	.	.	.	.	.	.	.
16	IKFRRVKGHLFTGT	17	0.36%	12	.	.	.	R	.	.	.	.	.	.	.	.	.	T
17	IRFKQVKGHLLTGI	17	0.36%	11	.	R	.	.	Q	.	.	.	.	.	L	.	.	.
18	IRFKRVKGHLLTGI	15	0.32%	12	.	R	.	.	.	.	.	.	.	.	L	.	.	.
19	IKFKRVKGHLLTGT	14	0.30%	10	.	.	.	.	.	.	.	.	.	.	L	.	.	T
20	IRFKRAKGHLFTGI	11	0.23%	12	.	R	.	.	.	A	.	.	.	.	.	.	.	.
21	IRFKRVKGHLFTGT	11	0.23%	12	.	R	.	.	.	.	.	.	.	.	.	.	.	T
22	IKFKQAEGHLFTGI	10	0.21%	9	.	.	.	.	Q	A	E	.	.	.	.	.	.	.
23	IRFKQVKGHLFTGI	10	0.21%	12	.	R	.	.	Q	.	.	.	.	.	.	.	.	.
24	IKFKQVKGHLLTGT	9	0.19%	9	.	.	.	.	Q	.	.	.	.	.	L	.	.	T
25	IKFKQVKGHLLTVI	9	0.19%	10	.	.	.	.	Q	.	.	.	.	.	L	.	V	.
26	IKFKRAKGHLLTGI	9	0.19%	10	.	.	.	.	.	A	.	.	.	.	L	.	.	.
27	IKFKRVEGHLFTGT	8	0.17%	10	.	.	.	.	.	.	E	.	.	.	.	.	.	T
28	TKFKRVKGHFFTAI	8	0.17%	9	T	.	.	.	.	.	.	.	.	F	.	.	A	.
29	TKFKRVKGHLFTAI	8	0.17%	10	T	.	.	.	.	.	.	.	.	.	.	.	A	.
30	IKFKQVKGHFFIGI	7	0.15%	9	.	.	.	.	Q	.	.	.	.	F	.	I	.	.
31	IKFKRVEGHFFTGT	7	0.15%	9	.	.	.	.	.	.	E	.	.	F	.	.	.	T
32	IRFKRVEGHLFTGI	7	0.15%	12	.	R	.	.	.	.	E	.	.	.	.	.	.	.
33	TKFKRVKGHLLTAT	7	0.15%	8	T	.	.	.	.	.	.	.	.	.	L	.	A	T
34	IKFKRVKGHLLTVI	6	0.13%	11	.	.	.	.	.	.	.	.	.	.	L	.	V	.
35	IKFKRAKGHLFTAI	5	0.11%	10	.	.	.	.	.	A	.	.	.	.	.	.	A	.
36	IKFKRVEGHLLTGI	5	0.11%	10	.	.	.	.	.	.	E	.	.	.	L	.	.	.
37	IKFKRVKGHFFTAI	5	0.11%	10	.	.	.	.	.	.	.	.	.	F	.	.	A	.
38	IKFKRVKGHFFTGT	5	0.11%	10	.	.	.	.	.	.	.	.	.	F	.	.	.	T
39	IKFKRVKGHLFTGV	5	0.11%	12	.	.	.	.	.	.	.	.	.	.	.	.	.	V

© 2023 Zhao LP et al. *JAMA Network Open.*

eTable 7.Haplotype Frequencies Among Gamma Viruses

Gamma. Haplotype frequencies of core haplotypes associated with variant-specific polymutants, for those haplotypes with 0.1% haplotype frequency.

ID	Freq	%	#	NSP3 S370	NSP3 K977	NSP4 S184	NSP12 P323	NSP13 E341	Spike L18	Spike T20	Spike P26	Spike D138	Spike R190	Spike K417	Spike E484	Spike N501	Spike D614	Spike H655	Spike T1027	Spike V1176	NS3 S253	NS8 E92	N P80	N R203	N G204
1	36853	61.22%	21	**L**	**Q**	**S**	**L**	**D**	**F**	**N**	**S**	**Y**	**S**	**T**	**K**	**Y**	**G**	**Y**	**I**	**F**	**P**	**K**	**R**	**K**	**R**
2	7567	12.57%	22	.	.	N	.	.	.	.	.	.	.	.	.	.	.	.	.	.	.	.	.	.	.
3	1626	2.70%	20	.	.	.	.	.	.	.	.	.	R	.	.	.	.	.	.	.	.	.	.	.	.
4	1400	2.33%	19	.	.	.	.	.	.	.	.	.	.	.	.	.	.	.	.	.	.	.	.	R	G
5	1375	2.28%	20	.	.	.	.	.	.	.	.	D	.	.	.	.	.	.	.	.	.	.	.	.	.
6	1365	2.27%	20	.	.	.	.	.	.	.	.	.	.	K	.	.	.	.	.	.	.	.	.	.	.
7	1005	1.67%	19	.	.	.	.	.	.	.	.	.	.	.	E	N	.	.	.	.	.	.	.	.	.
8	324	0.54%	20	.	.	.	.	.	.	.	.	.	.	.	.	.	.	.	T	.	.	.	.	.	.
9	263	0.44%	20	.	.	.	.	.	.	.	.	.	.	.	.	.	.	.	.	.	.	.	.	R	.
10	259	0.43%	20	.	.	N	.	.	.	.	.	.	.	.	.	.	.	.	T	V	.	.	.	.	.
11	246	0.41%	18	.	.	.	.	E	.	T	.	.	.	.	.	.	.	.	.	.	.	E	.	.	.
12	230	0.38%	21	.	.	N	.	.	.	.	.	D	.	.	.	.	.	.	.	.	.	.	.	.	.
13	220	0.37%	21	.	.	N	.	.	.	.	.	.	.	K	.	.	.	.	.	.	.	.	.	.	.
14	205	0.34%	20	.	.	.	.	.	.	.	.	.	.	.	.	.	.	.	.	.	.	E	.	.	.
15	203	0.34%	21	.	.	N	.	.	.	.	.	.	.	.	.	.	.	.	T	.	.	.	.	.	.
16	181	0.30%	20	S	.	.	.	.	.	.	.	.	.	.	.	.	.	.	.	.	.	.	.	.	.
17	173	0.29%	17	.	.	.	.	.	.	.	.	.	.	.	E	N	.	.	.	.	.	.	.	R	G
18	166	0.28%	18	.	.	.	.	.	.	.	.	.	R	.	E	N	.	.	.	.	.	.	.	.	.
19	157	0.26%	20	.	.	.	.	.	.	.	.	.	.	.	E	.	.	.	.	.	.	.	.	.	.
20	137	0.23%	19	.	.	.	.	.	.	.	.	.	.	.	.	.	.	.	T	V	.	.	.	.	.
21	135	0.22%	20	.	.	.	.	.	.	.	.	.	.	.	.	.	.	.	.	V	.	.	.	.	.
22	133	0.22%	21	.	.	.	.	.	.	.	F	.	.	.	.	.	.	.	.	.	.	.	.	.	.
23	124	0.21%	20	.	.	.	.	.	.	.	.	.	.	.	.	N	.	.	.	.	.	.	.	.	.
24	122	0.20%	19	.	.	.	.	.	.	.	.	.	R	K	.	.	.	.	.	.	.	.	.	.	.
25	100	0.17%	16	.	.	.	.	.	L	T	P	D	R	.	.	.	.	.	.	.	.	.	.	.	.
26	91	0.15%	18	.	.	.	.	.	L	T	P	.	.	.	.	.	.	.	.	.	.	.	.	.	.
27	90	0.15%	20	.	K	.	.	.	.	.	.	.	.	.	.	.	.	.	.	.	.	.	.	.	.
28	90	0.15%	17	.	.	.	.	.	L	T	P	.	R	.	.	.	.	.	.	.	.	.	.	.	.
29	83	0.14%	18	.	.	.	.	.	.	.	.	.	R	.	.	.	.	.	.	.	.	.	.	R	G
30	72	0.12%	19	.	.	.	.	.	.	.	.	D	.	K	.	.	.	.	.	.	.	.	.	.	.
31	72	0.12%	20	.	.	.	.	.	.	.	.	.	.	.	.	.	.	H	.	.	.	.	.	.	.
32	68	0.11%	20	.	.	.	.	E	.	.	.	.	.	.	.	.	.	.	.	.	.	.	.	.	.
33	67	0.11%	20	.	.	.	.	.	.	.	.	.	.	.	.	.	.	.	.	.	S	.	.	.	.
34	66	0.11%	20	.	.	.	.	.	.	.	.	.	.	.	.	.	.	.	.	.	.	.	P	.	.
35	64	0.11%	20	.	.	N	.	.	.	.	.	.	.	.	E	N	.	.	.	.	.	.	.	.	.
36	62	0.10%	19	.	.	.	.	.	.	.	.	D	.	.	.	.	.	.	.	.	.	E	.	.	.

© 2023 Zhao LP et al. *JAMA Network Open.*

eTable 8. Haplotype Frequencies Among GH/490R Viruses

GH/490R. Haplotype frequencies of core haplotypes associated with variant-specific polymutants, for those haplotypes with 0.1% haplotype frequency.

ID	**Freq**	%	#	NSP2 P129	NSP2 G221	NSP2 E272	NSP3 L1301	NSP3 A1537	NSP4 S386	NSP4 R401	NSP4 T492	NSP6 V149	NSP13 Q586	NSP16 R216	Spike P9	Spike E96	Spike R190	Spike I210	Spike Y449	Spike F490	Spike N501	Spike D936	NS3 T32	NS3 A54	N D22	N E378
1	78	18.10%	20	L	G	G	F	S	F	H	I	A	Q	R	L	Q	S	T	N	R	Y	H	I	S	Y	Q
2	69	16.01%	19	.	.	.	.	.	.	.	.	.	.	.	.	.	.	.	.	.	.	.	.	A	.	.
3	37	8.58%	21	.	S	.	.	.	.	.	.	.	H	H	.	.	.	.	Y	.	.	.	.	A	.	.
4	35	8.12%	22	.	S	.	.	.	.	.	.	.	H	H	.	.	.	.	.	.	.	.	.	A	.	.
5	22	5.10%	18	.	.	.	.	.	.	.	.	.	.	.	.	.	.	.	.	.	.	.	.	A	D	.
6	17	3.94%	18	.	.	.	.	.	.	.	.	.	.	.	.	.	.	.	Y	.	.	.	.	A	.	.
7	13	3.02%	19	.	.	.	.	.	.	.	.	.	.	.	.	.	.	.	Y	.	.	.	.	.	.	.
8	12	2.78%	18	.	.	.	.	.	.	.	.	.	.	.	.	.	R	I	.	.	.	.	.	.	.	.
9	10	2.32%	9	.	.	E	L	A	S	R	T	V	.	.	.	.	.	I	.	S	.	D	.	A	.	E
10	9	2.09%	15	.	.	.	.	.	.	.	.	.	.	.	.	E	R	I	.	.	.	.	.	A	D	.
11	7	1.62%	17	.	.	.	.	.	.	.	.	.	.	.	.	.	R	I	.	.	.	.	.	A	.	.
12	6	1.39%	20	.	.	.	.	.	.	.	.	.	.	.	.	.	.	.	.	.	.	.	.	V	.	.
13	5	1.16%	8	.	.	E	L	A	S	R	T	V	.	.	.	.	.	I	Y	S	.	D	.	A	.	E
14	5	1.16%	16	.	.	.	.	A	.	.	.	.	.	.	.	.	R	I	.	.	.	.	.	A	.	.
15	5	1.16%	19	.	.	.	.	.	S	.	.	.	.	.	.	.	.	.	.	.	.	Y	.	.	.	.
16	4	0.93%	8	.	.	E	L	A	S	R	T	V	.	.	.	.	R	I	.	S	.	D	.	A	.	E
17	4	0.93%	10	.	.	E	L	A	S	R	T	V	.	.	.	.	.	I	.	S	.	D	.	.	.	E
18	4	0.93%	18	.	.	.	.	A	.	.	.	.	.	.	.	.	.	.	.	.	.	.	.	A	.	.
19	4	0.93%	19	.	.	.	.	A	.	.	.	.	.	.	.	.	.	.	.	.	.	.	.	.	.	.
20	4	0.93%	18	.	.	.	.	.	.	.	.	.	.	.	.	.	R	.	.	.	.	.	.	A	.	.
21	4	0.93%	21	.	S	.	.	.	.	.	.	.	H	H	.	.	R	.	.	.	.	.	.	A	.	.
22	3	0.70%	9	.	.	E	L	A	S	R	T	V	.	.	.	.	R	I	.	S	.	D	.	.	.	E
23	3	0.70%	16	.	.	.	.	.	.	.	.	.	.	.	.	E	R	I	.	.	.	.	.	.	D	.
24	3	0.70%	19	.	.	.	.	.	S	.	.	.	.	.	.	.	.	.	.	.	.	.	.	.	.	.
25	3	0.70%	18	.	.	.	L	A	.	.	.	.	H	H	.	.	.	.	Y	.	.	.	.	A	.	.
26	3	0.70%	20	.	S	.	.	.	.	.	.	.	H	H	.	.	R	I	.	.	.	.	.	A	.	.
27	2	0.46%	5	.	.	E	L	A	S	R	T	V	.	.	.	E	R	I	.	S	.	D	T	A	D	E
28	2	0.46%	17	.	.	.	.	.	.	.	.	.	.	.	.	E	.	.	.	.	.	.	.	A	D	.
29	2	0.46%	19	.	.	.	.	.	.	.	.	.	.	.	.	.	.	.	.	.	.	.	.	.	D	.
30	2	0.46%	17	.	.	.	L	A	.	.	.	.	.	.	.	.	.	.	.	.	.	.	.	A	.	.
31	2	0.46%	17	.	S	.	.	.	.	.	.	.	H	H	.	.	R	I	Y	F	N	.	.	A	.	.
32	2	0.46%	17	P	.	.	.	.	.	.	.	.	.	.	.	.	.	.	.	.	.	.	.	A	D	.
33	1	0.23%	7	.	.	E	L	A	S	R	T	V	.	.	P	.	R	I	.	S	.	D	.	A	.	E
34	1	0.23%	15	.	.	E	L	.	.	.	.	.	.	.	.	.	.	.	Y	.	.	.	.	A	.	E
35	1	0.23%	14	.	.	.	.	A	.	.	.	.	.	.	.	E	R	I	.	.	.	.	.	A	D	.
36	1	0.23%	14	.	.	.	.	A	.	.	.	.	.	.	.	.	R	I	.	.	.	.	.	A	D	E
37	1	0.23%	17	.	.	.	.	A	.	.	.	.	.	.	.	.	R	I	.	.	.	.	.	.	.	.
38	1	0.23%	17	.	.	.	.	A	.	.	.	.	.	.	.	.	.	I	.	.	.	.	.	A	.	.
39	1	0.23%	18	.	.	.	.	A	.	.	.	.	.	.	.	.	.	.	.	.	.	.	.	.	.	E
40	1	0.23%	17	.	.	.	.	A	.	.	.	.	.	.	.	.	.	.	Y	.	.	.	.	A	.	.

© 2023 Zhao LP et al. *JAMA Network Open.*

eTable 9. Haplotype Frequencies Among Iota Viruses

Iota. Haplotype frequencies of core haplotypes associated with variant-specific polymutants, for those haplotypes with 0.1% haplotype frequency.

ID	Freq	%	#	NSP2	NSP4	NSP12	NSP13	Spike	Spike	Spike	Spike	Spike	Spike	Spike	Spike	NS3	NS3	NS7a	NS8	N	N	N	N
				T85	L438	P323	Q88	L5	T95	D253	S477	E484	D614	A701	Q957	P42	Q57	L116	T11	P13	P199	S202	M234
1	10264	47.21%	15	**I**	**P**	**L**	**H**	**F**	**I**	**G**	**S**	**K**	**G**	**V**	**Q**	**L**	**H**	**L**	**I**	**P**	**L**	**S**	**I**
2	5329	24.51%	16	.	.	.	.	.	.	.	N	E	.	A	R	.	.	F	.	L	P	R	M
3	1395	6.42%	15	.	.	.	.	.	.	.	N	E	.	.	.	.	.	.	.	.	.	.	.
4	902	4.15%	14	.	.	.	.	.	.	.	.	E	.	.	.	.	.	.	.	.	.	.	.
5	296	1.36%	15	.	.	.	.	.	.	.	N	E	.	A	R	.	.	.	.	L	P	R	M
6	279	1.28%	14	.	.	.	.	.	.	.	.	.	.	A	.	.	.	.	.	.	.	.	.
7	178	0.82%	14	.	.	.	.	L	.	.	.	.	.	.	.	.	.	.	.	.	.	.	.
8	152	0.70%	15	.	.	.	.	.	.	.	N	E	.	A	.	.	.	F	.	L	P	R	M
9	105	0.48%	14	.	.	.	.	.	.	.	.	.	.	.	.	P	.	.	.	.	.	.	.
10	101	0.46%	14	.	.	.	Q	.	.	.	.	.	.	.	.	.	.	.	.	.	.	.	.
11	87	0.40%	14	.	.	.	.	.	.	.	.	.	.	.	.	.	.	.	T	.	.	.	.
12	84	0.39%	14	.	.	.	.	.	.	D	.	.	.	.	.	.	.	.	.	.	.	.	.
13	83	0.38%	14	.	.	.	.	.	.	.	N	E	.	A	.	.	.	.	.	.	.	.	.
14	77	0.35%	13	.	.	.	.	.	.	.	.	.	.	A	.	.	.	.	T	.	.	.	.
15	74	0.34%	15	.	.	.	.	.	.	.	.	E	.	A	R	.	.	F	.	L	P	R	M
16	72	0.33%	15	.	.	.	.	L	.	.	N	E	.	A	R	.	.	F	.	L	P	R	M
17	55	0.25%	13	.	.	.	.	.	.	.	N	E	.	A	.	.	.	F	T	.	P	R	M
18	53	0.24%	13	.	.	P	.	.	.	.	.	.	.	A	.	.	.	.	.	.	.	.	.
19	49	0.23%	13	.	.	.	.	L	.	.	.	E	.	.	.	.	.	.	.	.	.	.	.
20	46	0.21%	15	.	.	.	Q	.	.	.	N	E	.	A	R	.	.	F	.	L	P	R	M
21	43	0.20%	15	.	.	.	.	.	.	.	N	E	.	A	R	P	.	F	.	L	P	R	M
22	43	0.20%	16	.	.	.	.	.	.	.	.	.	.	.	.	.	.	F	.	.	.	.	.
23	41	0.19%	14	.	.	.	.	L	.	.	N	E	.	A	R	.	.	.	.	L	P	R	M
24	38	0.17%	14	.	.	.	.	.	.	.	.	E	.	A	R	.	.	.	.	L	P	R	M
25	37	0.17%	13	.	.	.	.	.	.	.	.	.	.	.	.	.	.	.	.	.	P	.	M
26	36	0.17%	16	.	.	.	.	.	.	.	.	.	.	.	.	.	.	.	.	L	.	.	.
27	33	0.15%	15	.	.	.	.	.	.	D	N	E	.	A	R	.	.	F	.	L	P	R	M
28	29	0.13%	14	.	L	.	.	.	.	.	.	.	.	.	.	.	.	.	.	.	.	.	.
29	29	0.13%	14	.	.	P	.	.	.	.	.	.	.	.	.	.	.	.	.	.	.	.	.
30	28	0.13%	15	.	.	.	.	.	.	.	N	E	.	A	R	.	.	F	T	L	P	R	M
31	26	0.12%	13	.	.	.	.	.	.	.	.	E	.	A	.	.	.	.	.	.	.	.	.
32	26	0.12%	13	.	.	.	Q	L	.	.	.	.	.	.	.	.	.	.	.	.	.	.	.
33	24	0.11%	13	.	.	.	.	.	.	D	.	E	.	.	.	.	.	.	.	.	.	.	.
34	24	0.11%	13	.	.	.	.	.	.	.	N	E	.	A	.	.	.	.	T	.	.	.	.
35	24	0.11%	13	.	.	.	.	L	.	.	.	.	.	.	.	.	.	.	T	.	.	.	.

© 2023 Zhao LP et al. *JAMA Network Open.*

eTable 10. Haplotype Frequencies Among Kappa Viruses

Kappa. Haplotype frequencies of core haplotypes associated with variant-specific polymutants, for those haplotypes with 0.1% haplotype frequency.

ID	Freq	%	#	NSP3 T749	NSP6 T77	NSP12 P323	NSP13 G206	NSP13 M429	NSP15 P65	NSP15 K259	NSP15 S261	Spike T95	Spike G142	Spike E154	Spike L452	Spike E484	Spike D614	Spike P681	Spike Q1071	Spike H1101	NS3 S26	M I82	NS7a V82	NS8 S69	N D3	N R203	N D377
1	679	17.36%	20	**I**	**A**	**L**	**C**	**I**	**P**	**R**	**A**	**I**	**D**	**K**	**R**	**Q**	**G**	**R**	**H**	**H**	**L**	**S**	**A**	**S**	**D**	**M**	**Y**
2	228	5.83%	21	.	.	.	.	.	S	.	.	.	.	.	.	.	.	.	.	.	.	.	.	.	.	.	.
3	165	4.22%	19	.	.	.	G	.	.	.	S	T	.	.	.	.	.	.	.	D	.	I	.	L	Y	.	.
4	145	3.71%	18	.	.	.	G	.	.	.	S	T	.	.	.	.	.	.	.	D	.	I	.	L	.	.	.
5	126	3.22%	18	.	.	.	.	.	.	.	.	.	G	.	.	.	.	.	Q	.	.	.	.	.	.	.	.
6	96	2.45%	15	.	.	.	G	.	.	.	S	T	G	.	.	.	.	.	Q	D	.	I	.	.	.	.	.
7	89	2.28%	19	.	.	.	.	.	.	.	.	.	.	E	.	.	.	.	.	.	.	.	.	.	.	.	.
8	78	1.99%	18	.	.	.	.	.	.	.	.	.	G	E	.	.	.	.	.	.	.	.	.	.	.	.	.
9	65	1.66%	19	.	.	.	.	.	.	.	.	.	G	.	.	.	.	.	.	.	.	.	.	.	.	.	.
10	65	1.66%	17	.	.	.	G	.	.	.	S	T	.	.	.	.	.	.	.	D	.	I	.	.	.	.	.
11	57	1.46%	17	.	.	.	.	.	.	.	.	T	G	E	.	.	.	.	.	.	.	.	.	.	.	.	.
12	57	1.46%	17	.	.	.	G	.	.	.	.	T	.	.	.	.	.	.	.	.	.	I	.	.	.	.	.
13	49	1.25%	16	.	.	.	G	.	.	.	S	T	G	.	.	.	.	.	Q	D	.	I	.	.	Y	.	.
14	40	1.02%	15	.	.	.	G	.	.	.	.	T	G	E	.	.	.	.	.	.	.	I	.	.	.	.	.
15	38	0.97%	14	.	.	.	G	.	.	.	S	T	G	E	.	.	.	.	Q	D	.	I	.	.	.	.	.
16	37	0.95%	18	.	.	.	.	.	.	.	.	T	G	.	.	.	.	.	.	.	.	.	.	.	.	.	.
17	34	0.87%	18	.	.	.	G	.	.	.	S	T	.	.	.	.	.	.	.	Y	.	I	.	L	.	.	.
18	34	0.87%	16	.	.	.	G	.	.	.	S	T	G	E	.	.	.	.	.	D	.	I	.	L	.	.	.
19	34	0.87%	18	.	.	.	G	.	.	.	S	T	G	.	.	.	.	.	.	D	.	I	.	L	Y	.	.
20	33	0.84%	15	.	.	.	G	.	.	.	.	T	G	.	.	.	.	.	Q	.	.	I	.	.	.	.	.
21	32	0.82%	16	.	.	.	G	.	.	.	S	T	G	.	.	.	.	.	.	D	.	I	.	.	.	.	.
22	29	0.74%	18	.	.	.	G	.	.	.	S	T	.	E	.	.	.	.	.	D	.	I	.	L	Y	.	.
23	26	0.66%	15	.	.	.	G	.	.	.	S	T	G	E	.	.	.	.	.	D	.	I	.	.	.	.	.
24	23	0.59%	17	.	.	.	G	.	.	.	S	T	G	.	.	.	.	.	.	D	.	I	.	L	.	.	.
25	23	0.59%	19	T	.	.	.	.	.	.	.	.	.	.	.	.	.	.	.	.	.	.	.	.	.	.	.
26	22	0.56%	17	.	.	.	.	.	.	.	.	.	G	E	.	.	.	.	Q	.	.	.	.	.	.	.	.
27	22	0.56%	20	.	.	.	.	.	S	.	.	.	.	E	.	.	.	.	.	.	.	.	.	.	.	.	.
28	21	0.54%	14	.	.	.	G	.	.	.	.	T	G	E	.	.	.	.	Q	.	.	I	.	.	.	.	.
29	21	0.54%	16	.	.	.	G	.	.	.	.	T	G	.	.	.	.	.	.	.	.	I	.	.	.	.	.
30	21	0.54%	17	.	.	.	G	.	.	.	S	T	G	E	.	.	.	.	.	D	.	I	.	L	Y	.	.
31	20	0.51%	16	.	.	.	G	.	.	.	S	T	G	E	.	.	.	.	.	D	.	I	.	.	Y	.	.
32	18	0.46%	19	.	.	.	.	.	.	.	.	.	V	S	.	.	.	.	Q	.	.	.	.	.	.	.	.
33	18	0.46%	19	.	.	.	.	.	S	.	.	.	G	E	.	.	.	.	.	.	.	.	.	.	.	.	.
34	18	0.46%	20	.	.	.	.	.	S	.	.	.	G	.	.	.	.	.	.	.	.	.	.	.	.	.	.
35	18	0.46%	20	T	.	.	.	.	S	.	.	.	.	.	.	.	.	.	.	.	.	.	.	.	.	.	.
36	17	0.43%	19	.	.	.	.	.	.	.	.	.	.	.	.	.	.	.	.	.	.	.	V	.	.	.	.
37	17	0.43%	19	.	.	.	.	.	.	.	.	.	.	.	.	.	.	.	Q	.	.	.	.	.	.	.	.
38	16	0.41%	17	.	.	.	G	.	.	.	S	T	.	E	.	.	.	.	.	D	.	I	.	L	.	.	.
39	15	0.38%	16	.	.	.	.	.	.	.	.	T	G	E	.	.	.	.	Q	.	.	.	.	.	.	.	.
40	15	0.38%	17	.	.	.	G	.	.	.	S	T	G	.	.	.	.	.	.	D	.	I	.	.	Y	.	.

© 2023 Zhao LP et al. *JAMA Network Open.*

eTable 11. Haplotype Frequencies Among Lambda Viruses Lambda

Haplotype frequencies of core haplotypes associated with variant-specific polymutants, for those haplotypes with 0.1% haplotype frequency.

ID	Freq	%	#	NSP3	NSP3	NSP3	NSP4	NSP4	NSP5	NSP12	Spike	Spike	Spike	Spike	Spike	Spike	Spike	NS3	N	N	N	N	N
				T428	P1469	F1569	L438	T492	G15	P323	G75	T76	D253	L452	F490	D614	T859	A110	P13	A119	R203	G204	G214
1	2885	56.57%	18	**I**	**S**	**V**	**P**	**I**	**S**	**L**	**V**	**I**	**N**	**Q**	**S**	**G**	**N**	**A**	**L**	**A**	**K**	**R**	**C**
2	472	9.25%	17	.	.	.	.	.	.	.	.	.	D	.	.	.	.	.	.	.	.	.	.
3	401	7.86%	17	.	.	.	.	.	G	.	.	.	.	.	.	.	.	.	.	.	.	.	.
4	323	6.33%	20	.	.	.	.	.	.	.	.	.	.	.	.	.	.	S	.	P	.	.	.
5	137	2.69%	16	.	.	.	.	.	.	.	G	T	.	.	.	.	.	.	.	.	.	.	.
6	104	2.04%	19	.	.	.	.	.	.	.	.	.	D	.	.	.	.	S	.	P	.	.	.
7	54	1.06%	19	.	.	.	.	.	.	.	.	.	.	.	.	.	.	.	.	P	.	.	.
8	40	0.78%	16	.	.	.	.	.	G	.	.	.	D	.	.	.	.	.	.	.	.	.	.
9	36	0.71%	15	.	.	.	.	.	.	.	G	T	D	.	.	.	.	.	.	.	.	.	.
10	30	0.59%	17	.	.	.	.	.	.	.	.	.	.	.	.	.	.	.	P	.	.	.	.
11	27	0.53%	15	.	.	.	.	.	.	.	.	.	D	.	.	.	.	.	.	.	R	G	.
12	23	0.45%	16	.	P	.	.	.	.	.	.	.	D	.	.	.	.	.	.	.	.	.	.
13	23	0.45%	18	.	.	.	.	.	.	.	.	.	D	.	.	.	.	.	.	P	.	.	.
14	23	0.45%	16	.	.	.	.	.	.	.	.	.	.	.	.	.	.	.	.	.	R	G	.
15	20	0.39%	18	.	.	.	.	.	.	.	.	.	.	.	.	.	.	.	.	.	.	.	S
16	18	0.35%	15	.	.	.	.	.	G	.	G	T	.	.	.	.	.	.	.	.	.	.	.
17	17	0.33%	17	.	.	.	.	.	.	.	.	.	.	.	.	.	.	.	.	.	R	.	.
18	17	0.33%	17	.	.	.	.	.	.	.	.	.	.	.	.	.	T	.	.	.	.	.	.
19	12	0.24%	17	.	P	.	.	.	.	.	.	.	.	.	.	.	.	.	.	.	.	.	.
20	11	0.22%	17	.	.	.	.	.	.	.	.	.	.	.	F	.	.	.	.	.	.	.	.
21	11	0.22%	18	.	.	.	.	.	.	.	.	.	.	.	.	.	.	.	F	.	.	.	.
22	10	0.20%	14	.	.	.	.	.	G	.	G	T	D	.	.	.	.	.	.	.	.	.	.
23	9	0.18%	16	.	.	.	.	.	.	.	.	.	.	L	F	.	.	.	.	.	.	.	.
24	7	0.14%	14	.	P	.	.	.	.	.	.	.	D	L	F	.	.	.	.	.	.	.	.
25	7	0.14%	19	.	.	.	.	.	G	.	.	.	.	.	.	.	.	S	.	P	.	.	.
26	7	0.14%	17	.	.	.	.	.	.	.	G	.	.	.	.	.	.	.	.	.	.	.	.
27	6	0.12%	15	.	P	.	.	.	.	.	.	.	.	L	F	.	.	.	.	.	.	.	.
28	6	0.12%	17	.	.	.	.	.	.	.	.	.	D	.	.	.	.	.	.	.	.	.	S
29	6	0.12%	14	.	.	.	.	.	.	.	.	.	D	.	.	.	.	.	.	.	R	G	G
30	6	0.12%	19	.	.	.	.	.	.	.	.	.	.	.	.	.	.	.	.	S	.	.	.
31	6	0.12%	19	.	.	.	.	.	.	.	.	.	.	.	.	.	.	S	.	.	.	.	.

© 2023 Zhao LP et al. *JAMA Network Open.*

eTable 12. Haplotype Frequencies Among Mu Viruses Mu

Haplotype frequencies of core haplotypes associated with variant-specific polymutants, for those haplotypes with 0.1% haplotype frequency.

ID	Freq	%	#	NSP3 T237	NSP3 T720	NSP3 N1329	NSP4 T189	NSP4 T492	NSP6 Q160	NSP12 P323	NSP12 Y521	NSP13 E261	NSP13 P419	NSP13 P491	NSP15 S261	Spike T95	Spike Y144	Spike Y145	Spike R346	Spike E484	Spike N501	Spike D614	Spike P681	Spike D950	NS3 I20	NS3 Q57	NS3 L106	NS3 V256	NS3 N257	NS3 V259	NS8 T11	NS8 P38	NS8 S67	N T205
1	1406	17.96%	21	**A**	**I**	**N**	**T**	**I**	**R**	**L**	**Y**	**E**	**S**	**P**	**S**	**I**	**S**	**N**	**K**	**K**	**Y**	**G**	**H**	**N**	**I**	**H**	**L**	**I**	**N**	**V**	**K**	**S**	**F**	**I**
2	667	8.52%	27	.	.	D	I	.	.	.	.	.	.	S	L	.	.	.	.	.	.	.	.	.	.	.	.	.	H	L	.	.	.	.
3	561	7.16%	24	.	.	.	.	.	.	.	C	D	.	.	.	.	.	.	.	.	.	.	.	.	M	.	.	.	.	.	.	.	.	.
4	482	6.16%	22	.	.	.	.	.	.	.	.	.	.	.	.	.	.	.	.	.	.	.	.	.	.	.	F	.	.	.	.	.	.	.
5	405	5.17%	22	.	.	.	.	.	.	.	.	.	.	S	.	.	.	.	.	.	.	.	.	.	.	.	.	.	.	.	.	.	.	.
6	238	3.04%	19	.	.	.	.	.	.	.	.	.	.	.	.	.	Y	Y	.	.	.	.	.	.	.	.	.	.	.	.	.	.	.	.
7	224	2.86%	20	.	.	.	.	.	.	.	.	.	.	.	.	.	Y	.	.	.	.	.	.	.	.	.	.	.	.	.	.	.	.	.
8	210	2.68%	22	.	.	.	.	.	.	.	C	.	.	.	.	.	.	.	.	.	.	.	.	.	.	.	.	.	.	.	.	.	.	.
9	140	1.79%	26	.	.	D	I	.	.	.	.	.	.	S	L	.	.	.	.	.	.	.	.	.	.	.	.	.	H	L	.	.	S	.
10	125	1.60%	20	.	.	.	.	.	.	.	.	.	.	.	.	.	.	.	.	.	.	.	.	B	.	.	.	.	.	.	.	.	S	.
11	110	1.40%	21	.	.	.	.	.	.	.	C	.	.	.	.	.	.	.	.	.	.	.	.	B	.	.	.	.	.	.	.	.	S	.
12	96	1.23%	25	.	.	.	.	.	.	.	C	D	.	.	L	.	.	.	.	.	.	.	.	.	M	.	.	.	.	.	.	.	.	.
13	92	1.17%	20	.	.	.	.	.	.	.	.	.	.	.	.	.	.	.	.	.	.	.	.	.	.	.	.	.	.	.	.	.	S	.
14	81	1.03%	21	.	.	.	.	.	.	.	.	.	.	S	.	.	N	Y	.	.	.	.	.	.	.	.	.	.	.	.	.	.	.	.
15	75	0.96%	21	.	.	.	.	.	.	.	.	.	.	.	.	.	Y	.	.	.	.	.	.	.	.	.	F	.	.	.	.	.	.	.
16	64	0.82%	21	.	.	.	.	.	.	.	.	.	.	S	.	.	Y	.	.	.	.	.	.	.	.	.	.	.	.	.	.	.	.	.
17	62	0.79%	20	.	.	.	.	.	.	.	.	.	.	.	.	.	T	Y	.	.	.	.	.	.	.	.	.	.	.	.	.	.	.	.
18	59	0.75%	21	.	.	.	.	.	.	.	.	.	.	S	.	.	.	.	.	.	.	.	.	B	.	.	.	.	.	.	.	.	S	.
19	57	0.73%	23	.	.	.	.	.	.	.	C	D	.	.	.	.	.	.	.	.	.	.	.	B	M	.	.	.	.	.	.	.	S	.
20	49	0.63%	21	.	.	.	.	.	.	.	.	.	.	.	.	.	.	.	.	.	.	.	.	B	.	.	F	.	.	.	.	.	S	.
21	44	0.56%	23	.	.	.	.	.	.	.	C	D	.	.	.	.	Y	.	.	.	.	.	.	.	M	.	.	.	.	.	.	.	.	.
22	42	0.54%	23	.	.	.	.	.	.	.	C	D	.	.	.	.	.	.	.	.	.	.	.	.	M	.	.	.	.	.	.	.	S	.
23	42	0.54%	18	.	.	.	.	.	.	.	.	.	.	.	.	T	Y	Y	.	.	.	.	.	.	.	.	.	.	.	.	.	.	.	.
24	37	0.47%	19	.	.	.	.	.	.	.	.	.	.	.	.	.	.	.	.	E	N	.	.	.	.	.	.	.	.	.	.	.	.	.
25	37	0.47%	21	.	.	.	.	.	.	.	.	.	.	.	.	.	.	.	.	.	.	.	.	.	.	.	F	.	.	.	.	.	S	.
26	36	0.46%	21	.	.	.	.	.	.	.	C	.	.	.	.	.	Y	.	.	.	.	.	.	.	.	.	.	.	.	.	.	.	.	.
27	31	0.40%	19	.	.	.	.	.	.	.	.	.	.	.	.	.	.	.	.	.	.	.	.	D	.	.	.	.	.	.	.	.	S	.
28	26	0.33%	22	.	.	.	.	.	.	.	C	D	.	.	.	.	Y	Y	.	.	.	.	.	.	M	.	.	.	.	.	.	.	.	.
29	26	0.33%	19	.	.	.	.	.	.	.	.	.	.	.	.	.	.	.	.	.	.	.	.	.	.	.	.	.	.	.	.	.	S	T
30	25	0.32%	21	.	.	.	.	.	.	.	.	.	.	S	.	.	T	Y	.	.	.	.	.	.	.	.	.	.	.	.	.	.	.	.
31	24	0.31%	18	.	.	.	.	.	.	.	.	.	.	.	.	.	Y	Y	.	.	.	.	.	.	.	.	.	V	.	.	.	.	.	.
32	24	0.31%	21	.	.	.	.	.	.	.	.	.	.	S	.	.	.	.	.	.	.	.	.	.	.	.	.	.	.	.	.	.	S	.
33	22	0.28%	26	.	.	D	I	.	.	.	.	.	.	S	.	.	.	.	.	.	.	.	.	.	.	.	.	.	H	L	.	.	.	.
34	21	0.27%	23	.	.	.	.	.	.	.	C	D	.	.	.	.	T	Y	.	.	.	.	.	.	M	.	.	.	.	.	.	.	.	.
35	21	0.27%	20	.	.	.	.	.	.	.	.	.	.	.	.	.	.	.	.	.	.	.	.	D	.	.	.	.	.	.	.	.	.	.
36	20	0.26%	23	.	.	.	.	.	.	.	C	D	.	.	.	.	.	.	.	.	.	.	.	.	.	.	.	.	.	.	.	.	.	.
37	20	0.26%	20	.	.	.	.	.	.	.	.	.	.	.	.	.	.	.	.	.	.	.	.	.	.	.	.	V	.	.	.	.	.	.
38	20	0.26%	20	.	.	.	.	.	.	P	.	.	.	.	.	.	.	.	.	.	.	.	.	.	.	.	.	.	.	.	.	.	.	.
39	19	0.24%	25	.	.	D	I	.	.	.	.	.	.	S	L	.	.	Y	.	.	.	.	.	.	.	.	.	.	H	.	.	.	.	.

© 2023 Zhao LP et al. *JAMA Network Open.*

eTable 13. Haplotype Frequencies Among Omicron Viruses

Omicron. Haplotype frequencies of core haplotypes associated with variant-specific polymutants, for those haplotypes with 0.1% haplotype frequency.

ID	Freq	%	#	NSP1 S135	NSP3 T24	NSP3 K38	NSP3 G489	NSP3 L1266	NSP3 A1892	NSP4 L264	NSP4 T327	NSP4 L438	NSP4 T492	NSP5 P132	NSP6 I189	NSP12 P323	NSP13 R392	NSP14 I42	NSP15 T112	Spike T19	Spike A27	Spike A67	Spike T95	Spike G142	Spike L212	Spike V213	Spike G339	Spike R346	Spike S371	Spike S373	Spike S375	Spike T376	Spike D405	Spike R408	Spike K417	Spike N440	Spike G446	Spike S477	Spike T478	Spike E484	Spike Q493	Spike G496	Spike Q498	Spike N501	Spike Y505	Spike T547	Spike D614	Spike H655	Spike N679	Spike P681	Spike N764	Spike D796	Spike N856	Spike Q954	Spike N969	Spike L981	NS3 T223	E T9	M D3	M Q19	M A63	N P13	N R203	N G204	N S413
1	269527	16.85%	46	S	T	R	G	I	T	L	T	L	I	H	V	L	R	V	T	T	A	V	I	D	I	V	D	K	L	P	F	T	D	R	N	K	S	N	K	A	R	S	R	Y	H	K	G	Y	K	H	K	Y	K	H	K	F	T	I	G	E	T	L	K	R	S
2	262537	16.41%	48	R	I	K	S	L	A	F	I	F	.	.	I	.	C	.	I	I	S	A	T	.	L	G	.	R	F	.	.	A	N	S	.	.	G	.	.	.	.	G	.	.	.	T	.	.	.	.	.	.	N	.	.	L	I	.	D	.	.	.	.	.	R
3	189880	11.87%	45	.	.	.	.	.	.	.	.	.	.	.	.	.	.	.	.	.	.	.	.	.	.	.	.	R	.	.	.	.	.	.	.	.	.	.	.	.	.	.	.	.	.	.	.	.	.	.	.	.	.	.	.	.	.	.	.	.	.	.	.	.	.
4	59561	3.72%	42	.	.	.	.	.	.	.	.	.	.	.	.	.	.	.	.	.	.	.	.	.	.	.	.	R	.	.	.	.	.	.	K	N	G	.	.	.	.	.	.	.	.	.	.	.	.	.	.	.	.	.	.	.	.	.	.	.	.	.	.	.	.
5	41552	2.60%	43	.	.	.	.	.	.	.	.	.	.	.	.	.	.	.	.	.	.	.	.	.	.	.	.	.	.	.	.	.	.	.	K	N	G	.	.	.	.	.	.	.	.	.	.	.	.	.	.	.	.	.	.	.	.	.	.	.	.	.	.	.	.
6	33570	2.10%	41	.	.	.	.	.	.	.	.	.	.	.	.	.	.	.	.	.	.	.	.	.	.	.	G	R	S	S	S	.	.	.	.	.	.	.	.	.	.	.	.	.	.	.	.	.	.	.	.	.	.	.	.	.	.	.	.	.	.	.	.	.	.
7	28398	1.77%	41	.	.	.	.	.	.	.	.	.	.	.	.	.	.	.	.	.	.	.	.	.	.	.	.	R	.	.	.	.	.	.	K	N	G	.	.	.	.	.	.	.	.	.	.	.	.	.	N	.	.	.	.	.	.	.	.	.	.	.	.	.	.
8	20799	1.30%	47	R	I	K	S	L	A	F	I	F	.	.	I	.	C	.	.	I	S	A	T	.	L	G	.	R	F	.	.	A	N	S	.	.	G	.	.	.	.	G	.	.	.	T	.	.	.	.	.	.	N	.	.	L	I	.	D	.	.	.	.	.	R
9	14333	0.90%	42	.	.	.	.	.	.	.	.	.	.	.	.	.	.	.	.	.	.	.	.	.	.	.	.	.	.	.	.	.	.	.	K	N	G	.	.	.	.	.	.	.	.	.	.	.	.	.	N	.	.	.	.	.	.	.	.	.	.	.	.	.	.
10	14131	0.88%	48	R	I	K	S	L	A	F	I	F	.	.	I	.	C	.	I	I	S	A	T	.	L	G	.	R	F	.	.	A	N	S	.	.	G	.	.	.	.	G	.	.	.	T	.	.	.	.	.	.	N	.	.	L	I	.	D	Z	.	.	.	.	R
11	13166	0.82%	47	R	I	K	S	L	A	F	I	F	.	.	I	.	C	.	I	I	.	A	T	.	L	G	.	R	F	.	.	A	N	S	.	.	G	.	.	.	.	G	.	.	.	T	.	.	.	.	.	.	N	.	.	L	I	.	D	.	.	.	.	.	R
12	11981	0.75%	40	.	.	.	.	.	.	.	.	.	.	.	.	.	.	I	.	.	.	.	.	.	.	.	.	R	.	.	.	.	.	.	K	N	G	.	.	.	.	.	.	.	.	.	.	.	.	.	N	.	.	.	.	.	.	.	.	.	.	.	.	.	.
13	10932	0.68%	44	.	.	.	.	.	.	.	.	.	.	.	.	.	.	.	.	.	.	.	.	.	.	.	.	R	.	.	.	.	.	.	K	.	.	.	.	.	.	.	.	.	.	.	.	.	.	.	.	.	.	.	.	.	.	.	.	.	.	.	.	.	.
14	10375	0.65%	40	R	I	K	S	L	A	F	I	F	.	.	I	.	C	.	I	I	S	A	T	.	L	G	.	R	F	.	.	A	N	S	.	N	G	S	T	E	Q	G	Q	N	Y	T	.	.	.	.	.	.	N	.	.	L	I	.	D	.	.	.	.	.	R
15	9726	0.61%	30	.	.	.	.	.	.	.	.	.	.	.	.	.	.	.	.	.	.	.	.	.	L	.	.	R	S	S	S	.	.	.	K	N	G	S	T	E	Q	G	Q	N	Y	.	.	.	.	.	.	.	.	.	.	.	.	.	.	.	.	.	.	.	.
16	9372	0.59%	47	R	I	K	S	L	A	F	I	F	.	.	I	.	C	.	I	I	S	A	T	.	L	G	.	R	F	.	.	A	N	S	.	N	G	.	.	.	.	G	.	.	.	T	.	.	.	.	.	.	N	.	.	L	I	.	D	.	.	.	.	.	R
17	8908	0.56%	45	.	.	.	.	.	.	.	.	.	.	.	.	.	.	.	.	.	.	.	.	.	.	.	.	.	.	.	.	.	.	.	K	.	.	.	.	.	.	.	.	.	.	.	.	.	.	.	.	.	.	.	.	.	.	.	.	.	.	.	.	.	.
18	7726	0.48%	44	.	.	.	.	.	.	.	.	.	.	.	.	.	.	.	.	.	.	.	.	.	.	.	.	R	.	.	.	.	.	.	.	.	.	.	.	.	.	.	.	.	.	.	.	.	.	.	.	.	.	.	.	.	.	.	.	.	.	P	.	.	.
19	7324	0.46%	40	.	.	.	.	.	.	.	.	.	.	.	.	.	.	.	.	.	.	.	T	.	.	.	.	R	.	.	.	.	.	.	.	.	.	.	.	.	.	G	Q	N	Y	.	.	.	.	.	.	.	.	.	.	.	.	.	.	.	.	.	.	.	.
20	6598	0.41%	41	.	.	.	.	.	.	.	.	.	.	.	.	.	.	.	.	.	.	.	T	.	.	.	.	.	.	.	.	.	.	.	.	.	.	.	.	.	.	G	Q	N	Y	.	.	.	.	.	.	.	.	.	.	.	.	.	.	.	.	.	.	.	.
21	6484	0.41%	31	.	.	.	.	.	.	.	.	.	.	.	.	.	.	.	.	.	.	.	.	.	L	.	.	.	S	S	S	.	.	.	K	N	G	S	T	E	Q	G	Q	N	Y	.	.	.	.	.	.	.	.	.	.	.	.	.	.	.	.	.	.	.	.
22	6296	0.39%	39	.	.	.	.	.	.	.	.	.	.	.	.	.	.	.	.	.	.	.	T	G	.	.	.	R	.	.	.	.	.	.	.	.	.	.	.	.	.	G	Q	N	Y	.	.	.	.	.	.	.	.	.	.	.	.	.	.	.	.	.	.	.	.
23	5886	0.37%	40	.	.	.	.	.	.	.	.	.	.	.	.	.	.	.	.	.	.	.	T	G	.	.	.	.	.	.	.	.	.	.	.	.	.	.	.	.	.	G	Q	N	Y	.	.	.	.	.	.	.	.	.	.	.	.	.	.	.	.	.	.	.	.
24	5224	0.33%	45	R	I	K	S	L	A	F	I	F	.	.	I	.	C	.	I	I	S	A	T	.	L	G	.	R	F	.	.	A	N	S	.	.	G	.	.	.	.	G	Q	N	Y	T	.	.	.	.	.	.	N	.	.	L	I	.	D	.	.	.	.	.	R
25	5081	0.32%	29	.	.	.	.	.	.	.	.	.	.	.	.	.	.	.	.	.	.	.	.	.	L	.	G	R	S	S	S	.	.	.	K	N	G	S	T	E	Q	G	Q	N	Y	.	.	.	.	.	.	.	.	.	.	.	.	.	.	.	.	.	.	.	.
26	4705	0.29%	45	.	.	.	.	.	.	.	.	.	.	.	.	.	.	.	.	.	.	.	.	.	.	.	.	.	.	.	.	.	.	.	.	.	.	.	.	.	.	.	.	.	.	.	.	.	.	.	.	.	.	.	.	.	.	.	.	.	.	P	.	.	.
27	4598	0.29%	41	.	.	.	.	.	.	.	.	.	.	.	.	.	.	I	.	.	.	.	.	.	.	.	.	R	.	.	.	.	.	.	K	N	G	.	.	.	.	.	.	.	.	.	.	.	.	.	.	.	.	.	.	.	.	.	.	.	.	.	.	.	.
28	4446	0.28%	47	R	I	K	S	L	A	F	I	F	.	.	I	.	C	.	I	I	S	A	T	.	L	G	.	R	F	.	.	A	N	.	.	.	G	.	.	.	.	G	.	.	.	T	.	.	.	.	.	.	N	.	.	L	I	.	D	.	.	.	.	.	R
29	4275	0.27%	42	R	I	K	S	L	A	F	I	F	.	.	I	.	C	.	.	I	S	A	T	.	L	G	G	R	S	S	S	.	N	S	.	.	G	.	.	.	.	G	.	.	.	T	.	.	.	.	.	.	N	.	.	L	I	.	D	.	.	.	.	.	R
30	4047	0.25%	40	R	I	K	S	L	A	F	I	F	.	.	I	.	C	.	I	I	.	A	T	.	L	G	.	R	F	.	.	.	.	.	.	.	G	.	.	.	Q	G	Q	N	Y	T	.	.	.	.	.	.	N	.	.	L	I	.	D	.	.	.	.	.	R
31	4018	0.25%	47	R	I	K	S	L	A	F	I	.	.	.	I	.	C	.	I	I	S	A	T	.	L	G	.	R	F	.	.	A	N	S	.	.	G	.	.	.	.	G	.	.	.	T	.	.	.	.	.	.	N	.	.	L	I	.	D	.	.	.	.	.	R
32	3955	0.25%	47	.	I	K	S	L	A	F	I	F	.	.	I	.	C	.	I	I	S	A	T	.	L	G	.	R	F	.	.	A	N	S	.	.	G	.	.	.	.	G	.	.	.	T	.	.	.	.	.	.	N	.	.	L	I	.	D	.	.	.	.	.	R
33	3628	0.23%	41	.	.	.	.	.	.	.	.	.	.	.	.	.	.	I	.	.	.	.	.	.	.	.	.	.	.	.	.	.	.	.	K	N	G	.	.	.	.	.	.	.	.	.	.	.	.	.	N	.	.	.	.	.	.	.	.	.	.	.	.	.	.
34	3602	0.23%	44	.	.	.	.	.	.	.	.	.	.	.	.	.	.	.	.	.	.	.	.	.	.	.	.	R	.	.	.	.	.	.	.	.	.	.	.	.	.	.	.	.	.	.	.	.	.	.	.	.	.	.	.	.	.	.	D	.	.	.	.	.	.
35	3455	0.22%	45	.	.	.	.	.	.	.	.	.	.	.	.	.	.	.	.	.	.	.	.	.	L	.	.	.	.	.	.	.	.	.	.	.	.	.	.	.	.	.	.	.	.	.	.	.	.	.	.	.	.	.	.	.	.	.	.	.	.	.	.	.	.
36	3438	0.21%	44	.	.	.	.	.	.	.	.	.	.	.	.	.	.	.	.	.	.	.	.	G	.	.	.	.	.	.	.	.	.	.	K	.	.	.	.	.	.	.	.	.	.	.	.	.	.	.	.	.	.	.	.	.	.	.	.	.	.	.	.	.	.
37	3326	0.21%	42	.	.	K	.	.	.	.	.	.	.	P	.	.	.	.	.	.	.	.	.	.	.	.	.	.	.	.	.	.	.	.	K	.	.	.	.	.	.	.	.	.	.	.	.	.	.	.	.	.	.	.	.	.	.	.	D	.	.	.	.	.	.
38	3277	0.20%	47	R	I	K	S	L	A	F	I	F	.	.	I	.	C	.	I	I	S	A	T	G	L	G	.	R	F	.	.	A	N	S	.	.	G	.	.	.	.	G	.	.	.	T	.	.	.	.	.	.	N	.	.	L	I	.	D	Z	.	.	.	.	R
39	3264	0.20%	35	.	.	.	.	.	.	.	.	.	.	.	.	.	.	.	.	.	.	.	.	.	L	.	.	.	.	.	.	.	.	.	.	N	G	S	T	E	Q	G	Q	N	Y	.	.	.	.	.	.	.	.	.	.	.	.	.	.	.	.	.	.	.	.

© 2023 Zhao LP et al. *JAMA Network Open.*

eTable 14. Haplotype Frequencies Among Theta Viruses

Theta. Haplotype frequencies of core haplotypes associated with variant-specific polymutants, for those haplotypes with 0.1% haplotype frequency.

ID	Freq	%	#	NSP2 Y316	NSP2 G339	NSP2 A419	NSP3 D736	NSP3 S1807	NSP4 T204	NSP4 D217	NSP4 L438	NSP6 D112	NSP7 L71	NSP12 P323	NSP13 L280	NSP13 A368	NSP14 K155	Spike P9	Spike E484	Spike N501	Spike T573	Spike V1176	NS3 A23	NS8 K2	NS8 K68	N R203	N G204	N T391
1	123	37.27%	15	Y	G	A	G	F	T	N	P	E	F	L	F	V	K	P	K	Y	T	F	A	Q	K	K	R	T
2	53	16.06%	20	C	.	V	.	.	.	.	.	.	.	.	.	.	R	L	.	.	.	.	.	.	.	.	.	I
3	19	5.76%	14	.	.	.	.	.	.	.	.	.	.	.	L	.	.	.	.	.	.	.	.	.	.	.	.	.
4	14	4.24%	19	C	.	V	.	.	.	.	.	.	.	.	L	.	R	L	.	.	.	.	.	.	.	.	.	I
5	11	3.33%	18	.	S	.	.	.	.	.	L	.	.	.	.	.	.	.	.	.	I	.	S	.	E	.	.	.
6	9	2.73%	13	.	.	.	.	.	.	.	.	.	.	.	.	.	.	.	E	N	.	.	.	.	.	.	.	.
7	7	2.12%	16	.	.	.	.	.	.	.	.	.	.	.	.	.	.	L	.	.	.	.	.	.	.	.	.	.
8	6	1.82%	19	.	S	.	.	.	.	.	.	.	.	.	.	.	.	.	.	.	I	.	S	.	E	.	.	.
9	5	1.52%	16	.	.	.	.	.	I	.	.	.	.	.	.	.	.	.	.	.	.	.	.	.	.	.	.	.
10	5	1.52%	13	.	.	.	.	S	.	D	.	.	.	.	.	.	.	.	.	.	.	.	.	.	.	.	.	.
11	3	0.91%	11	.	.	.	.	.	.	.	.	.	.	.	.	.	.	.	E	N	.	.	.	.	.	R	G	.
12	2	0.61%	13	.	.	.	.	.	.	.	L	.	.	.	.	.	.	.	.	.	.	V	.	.	.	.	.	.
13	2	0.61%	14	.	.	.	.	.	.	.	.	.	.	.	.	.	.	.	E	.	.	.	.	.	.	.	.	.
14	2	0.61%	13	.	.	.	.	.	.	.	.	.	.	.	.	.	.	.	.	.	.	.	.	.	.	R	G	.
15	2	0.61%	16	.	.	.	.	.	.	.	.	.	.	.	.	.	.	S	.	.	.	.	.	.	.	.	.	.
16	2	0.61%	15	.	.	.	.	.	.	.	.	.	.	.	L	.	.	L	.	.	.	.	.	.	.	.	.	.
17	2	0.61%	10	.	.	.	.	.	.	.	.	.	.	.	L	.	.	.	E	N	.	.	.	.	.	R	G	.
18	2	0.61%	16	.	S	.	.	.	.	.	.	.	.	.	.	.	.	.	.	.	.	.	.	.	.	.	.	.
19	2	0.61%	16	.	S	.	.	.	.	.	.	.	.	.	L	.	.	.	E	N	I	.	S	.	E	.	.	.
20	1	0.30%	10	C	.	.	D	.	.	.	L	.	.	.	L	.	R	.	E	N	.	.	.	.	.	R	G	.
21	1	0.30%	16	C	.	V	D	.	.	.	.	D	.	P	L	.	R	L	.	.	.	.	.	.	.	.	.	I
22	1	0.30%	12	C	.	V	.	.	.	D	L	D	.	P	L	A	R	L	E	N	.	.	.	.	.	.	.	I
23	1	0.30%	16	C	.	V	.	.	.	D	.	.	.	P	L	A	R	L	.	.	.	.	.	.	.	.	.	I
24	1	0.30%	13	C	.	V	.	.	.	.	L	.	.	.	.	A	R	.	E	N	.	V	.	.	.	.	.	.
25	1	0.30%	19	C	.	V	.	.	.	.	L	.	.	.	.	.	R	L	.	.	.	.	.	.	.	.	.	I
26	1	0.30%	18	C	.	V	.	.	.	.	.	.	.	.	.	.	R	L	E	N	.	.	.	.	.	.	.	I
27	1	0.30%	19	C	.	V	.	.	.	.	.	.	.	.	.	.	R	L	E	.	.	.	.	.	.	.	.	I
28	1	0.30%	19	C	.	V	.	.	.	.	.	.	.	.	.	.	R	L	.	N	.	.	.	.	.	.	.	I
29	1	0.30%	18	C	.	V	.	.	.	.	.	.	.	.	.	.	R	L	.	.	.	.	.	.	.	R	G	I
30	1	0.30%	18	C	.	V	.	.	.	.	.	.	.	.	L	A	R	L	.	.	.	.	.	.	.	.	.	I
31	1	0.30%	17	C	.	V	.	.	.	.	.	.	.	.	L	.	R	L	E	N	.	.	.	.	.	.	.	I
32	1	0.30%	18	C	.	V	.	.	.	.	.	.	L	.	.	.	R	.	.	.	.	.	.	.	.	.	.	I
33	1	0.30%	17	C	.	V	.	.	.	.	.	.	L	.	L	A	R	L	.	.	.	.	.	.	.	.	.	I
34	1	0.30%	11	.	.	.	D	.	.	.	L	.	.	.	.	.	.	.	E	N	.	.	.	.	.	.	.	.
35	1	0.30%	9	.	.	.	D	.	.	.	L	.	.	.	.	.	.	.	E	N	.	.	.	.	.	R	G	.
36	1	0.30%	8	.	.	.	D	.	.	.	L	.	.	.	L	.	.	.	E	N	.	.	.	.	.	R	G	.
37	1	0.30%	9	.	.	.	D	.	.	.	.	D	.	P	L	.	.	.	E	N	.	.	.	.	.	.	.	.
38	1	0.30%	12	.	.	.	D	.	.	.	.	.	.	.	.	.	.	.	E	N	.	.	.	.	.	.	.	.
39	1	0.30%	11	.	.	.	D	.	.	.	.	.	.	.	.	.	.	.	E	N	.	.	.	.	.	R	.	.

© 2023 Zhao LP et al. *JAMA Network Open.*

eTable 15. Haplotype Frequencies Among Zeta Viruses

Zeta. Haplotype frequencies of core haplotypes associated with variant-specific polymutants, for those haplotypes with 0.1% haplotype frequency.

**ID**	**Freq**	**%**	**#**	NSP5 L205	NSP7 L71	NSP12 P323	Spike E484	Spike D614	Spike V1176	N A119	N R203	N G204	N M234
1	2550	0.8736	10	**V**	**F**	**L**	**K**	**G**	**F**	**S**	**K**	**R**	**I**
2	85	0.0291	9	L	.	.	.	.	.	.	.	.	.
3	80	0.0274	9	.	.	.	E	.	.	.	.	.	.
4	21	0.0072	9	.	.	.	.	.	V	.	.	.	.
5	18	0.0062	9	.	.	.	.	.	.	A	.	.	.
6	17	0.0058	8	.	.	P	E	.	.	.	.	.	.
7	17	0.0058	9	.	.	P	.	.	.	.	.	.	.
8	14	0.0048	8	.	.	P	.	.	V	.	.	.	.
9	12	0.0041	7	.	.	.	.	.	.	.	R	G	M
10	10	0.0034	8	.	.	.	.	.	.	.	R	G	.
11	10	0.0034	9	.	.	.	.	.	.	.	R	.	.
12	9	0.0031	9	.	.	.	.	.	.	.	.	.	M
13	8	0.0027	8	.	.	.	E	.	.	.	.	.	M
14	8	0.0027	10	.	.	.	.	.	.	.	.	L	.
15	4	0.0014	8	.	.	.	.	.	.	A	.	.	M
16	4	0.0014	8	.	.	.	.	.	V	.	.	.	M
17	3	0.001	6	L	.	.	E	D	V	.	.	.	.
18	3	0.001	8	.	.	.	.	.	.	A	R	.	.

© 2023 Zhao LP et al. *JAMA Network Open.*

eTable 16. Concordance Analysis in the Training Set

Result from the concordance analysis of GISAID assigned variant (columns) with haplotype-based variant assigment (rows) in the training data set with 4,393,998 viruses (50% of all viruses at GISAID, downloaded on March 14, 2022).

Predction[1]	Alpha	Beta	Delta	Epsilon	Eta	Gamma	GH/490R	Iota	Kappa	Lambda	Mu	Omicron	Theta	Zeta	UA[4]	Sub-total
**Alpha**	573,599		4			3									2,497	576,103
**Beta**		8,021													90	8,111
**Delta**	1	1	2,120,806						6			2			1,486	2,122,302
**Epsilon**	1		9	35,883				1			3	1			145,822	181,720
**Eta**	1		2		4,695										33	4,731
**Gamma**			1			59,471									95	59,567
**GH/490R**							422									422
**Iota**	1	1						18,589							68	18,659
**Kappa**	1		2						3,796						48	3,847
**Lambda**						1				5,077					6	5,084
**Mu**				1							5,634				38	5,673
**Omicron**			2									1,487,867			704	1,488,573
**Theta**													148		1	149
**Zeta**						4				1			1	2,918	24,546	27,470
**MV[2]**	385	12,940	19,917	11	16	658	8	3,140	43	9	2,193	112,137	181	1	7,633	159,272
**UP[3]**	446	97	1,702	53	3	63	1	12	66	13	2	1,333		1	360,335	364,127
**Sub-total**	574,435	21,060	2,142,445	35,948	4,714	60,200	431	21,742	3,911	5,100	7,832	1,601,340	330	2,920	543,402	5,025,810

[1]Posterial probability threshold=0.99; [2]MV for mixture of variants; [3]UP for unpredictable variant; [4]UC for unclassified variant

© 2023 Zhao LP et al. *JAMA Network Open.*

eTable 17. Concordance Analysis in the Validation Set

Result from the concordance analysis of GISAID assigned variant (columns) with haplotype-based variant assigment (rows) in the training data set with 4,393,998 viruses (50% of all viruses at GISAID, downloaded on March 14, 2022).

Predction[1]	Alpha	Beta	Delta	Epsilon	Eta	Gamma	GH/490R	Iota	Kappa	Lambda	Mu	Omicron	Theta	Zeta	UA[4]	Sub-total
**Alpha**	1,148,060	1	4			6			1						5,218	1,153,290
**Beta**		15,492													145	15,637
**Delta**	4		4,246,693			1			11			5			3,184	4,249,898
**Epsilon**	2	8	24	71,899		1		3			7	3			298,020	369,967
**Eta**	1	1	5		9,398										92	9,497
**Gamma**			1			118,898									193	119,092
**GH/490R**							8									8
**Iota**		2						37,951							143	38,096
**Kappa**			40						7,558						113	7,711
**Lambda**						2				10,058					16	10,076
**Mu**				1							7,348				60	7,409
**Omicron**			2							1		3,003,288			1,600	3,004,891
**Theta**													414		1	415
**Zeta**	3	5	3			12		4				2		5,886	53,014	58,929
**MV[2]**	732	26,350	35,585	56	54	1,399	879	5,315	90	12	8,518	194,988	211	1	18,028	292,218
**UP[3]**	825	122	3,248	84	3	86		14	98	24	5	3,232		1	706,744	714,486
**Sub-total**	1,149,627	41,981	4,285,605	72,040	9,455	120,405	887	43,287	7,758	10,095	15,878	3,201,518	625	5,888	1,086,571	10,051,620

[1]Posterial probability threshold=0.99; [2]MV for mixture of variants; [3]UP for unpredictable variant; [4]UA for unassigned variant

© 2023 Zhao LP et al. *JAMA Network Open.*

eTable 18. Concordance Analysis in the Prospective Set

Result from the concordance analysis of GISAID assigned variant (columns) with haplotype-based variant assigment (rows) in the prospective data set with 343,431 viruses, collected after March 14, 2022 and downloaded on May 18, 2022.

**Predction**[1]	Alpha	Delta	Epsilon	Eta	Lambda	Omicron	Zeta	**UA**[4]	Sub-total
Alpha	2					4			6
Delta		171				3			174
Epsilon			0			2		3	5
Eta				0				1	1
Lambda					1				1
Omicron						319,812		567	320,379
Zeta							0	1	1
**MV**[2]		5				22,072		30	22,107
**UP**[3]		4				1,699		524	2,227
**Sub-total**	2	180			1	343,592		1,126	344,901

© 2023 Zhao LP et al. *JAMA Network Open.*

eTable 19. Four Mixture Variants with Omicron-Delta

Four recombinants of Omicron with A) Delta, B) Alpha, C) Zeta and D) Epsilon variants, in which all observed recombinants include polymutants in their respective variant-specific core haplotypes and indicator of 1 and 2 corresponded to the variant (1) recombinaned with Omicron (2)

**Delta-Omicron**	Freq	NSP1 S135	NSP3 T24	NSP3 K38	NSP3 G489	NSP3 L1266	NSP3 A1892	NSP4 L264	NSP5 P132	NSP6 I189	NSP13 R392	NSP14 I42	NSP15 T112	Spike G339	Spike R346	Spike S371	Spike S373	Spike S375	Spike T376	Spike D405	Spike R408	Spike K417	Spike N440	Spike L452	Spike S477	Spike E484	Spike Q498	Spike N501	Spike Y505	Spike T547	Spike H655	Spike N679	Spike D796	Spike N856	Spike Q954	Spike N969	Spike L981	NS3 T223	E T9	M D3	M Q19	M A63	M I82	N P13	N G204	N S413
**Indicator**		2	2	2	2	2	2	2	2	2	2	2	2	2	2	2	2	2	2	2	2	2	2	1	2	2	2	2	2	2	2	2	2	2	2	2	2	2	2	2	2	2	1	2	2	2
1	1	R	I	.	S	.	.	F	H	.	C	V	I	D	.	F	P	F	A	N	S	N	K	R	N	A	R	Y	H	.	Y	.	.	.	.	.	.	I	I	.	E	T	.	L	R	R
2	1	.	I	.	S	.	.	F	H	.	C	V	I	D	.	F	P	F	A	N	S	N	K	R	N	A	R	Y	H	.	Y	.	.	.	.	.	.	I	I	.	E	T	.	L	R	R
3	1	.	.	R	.	I	T	.	H	V	.	V	.	D	K	L	P	F	.	.	.	.	.	R	N	.	R	Y	H	K	Y	K	Y	K	H	K	F	.	I	G	E	.	T	L	R	.

© 2023 Zhao LP et al. *JAMA Network Open.*

eTable 20. Four Mixture Variants with Omicron-Alpha

Four recombinants of Omicron with A) Delta, B) Alpha, C) Zeta and D) Epsilon variants, in which all observed recombinants include polymutants in their respective variant-specific core haplotypes and indicator of 1 and 2 corresponded to the variant (1) recombinaned with Omicron (2)

Alpha-Omicron	Freq	NSP1	NSP3	NSP3	NSP3	NSP3	NSP3	NSP3	NSP3	NSP4	NSP4	NSP4	NSP4	NSP5	NSP6	NSP13	NSP14	NSP15	Spike	Spike	Spike	Spike	Spike	Spike	Spike	Spike	Spike	Spike	Spike	Spike	Spike	Spike	Spike	Spike	Spike	Spike	Spike	Spike	Spike	Spike	Spike	Spike	Spike	Spike	Spike	Spike	Spike	Spike	Spike	Spike	Spike	Spike	Spike	Spike	NS3	E	M	M	M	NS8	N	N	N	N
		S135	T24	K38	T183	G489	L1266	I1412	A1892	L264	T327	L438	T492	P132	I189	R392	I42	T112	T19	A27	A67	T95	G142	L212	V213	G339	R346	S371	S373	S375	T376	D405	R408	K417	N440	G446	S477	T478	E484	Q493	G496	Q498	Y505	T547	H655	N679	T716	N764	D796	N856	Q954	N969	L981	S982	T223	T9	D3	Q19	A63	R52	D3	P13	S235	S413
**Indicator**		2	2	2	1	2	2	1	2	2	2	2	2	2	2	2	2	2	2	2	2	2	2	2	2	2	2	2	2	2	2	2	2	2	2	2	2	2	2	2	2	2	2	2	2	2	1	2	2	2	2	2	2	1	2	2	2	2	2	1	1	2	1	2
**1**	2	R	I	.	I	S	.	.	.	F	I	F	I	H	V	C	V	I	I	S	.	.	D	S	G	D	.	F	P	F	A	N	S	N	K	.	N	K	A	R	.	R	H	.	Y	K	.	K	Y	.	H	K	.	.	I	.	.	E	T	.	.	L	.	R
**2**	1	R	I	.	I	N	.	.	.	F	I	F	I	H	.	C	V	I	I	S	.	.	D	.	G	D	.	F	P	F	A	N	S	N	K	.	N	K	S	R	.	R	H	.	Y	K	.	K	Y	.	H	K	.	.	I	I	.	E	T	.	.	L	.	R
**3**	1	R	I	.	I	S	.	.	.	F	I	F	I	H	.	C	.	.	I	S	.	.	D	.	G	D	.	F	P	F	A	N	S	N	K	.	N	K	A	R	.	R	H	.	Y	K	.	K	.	.	.	K	.	.	I	I	.	E	T	.	.	L	.	R
**4**	1	R	I	.	I	S	.	.	.	F	I	F	I	H	.	C	V	I	I	S	.	.	D	.	G	D	.	Y	P	F	A	N	S	N	.	.	N	K	.	R	.	R	H	.	Y	K	.	K	Y	.	H	K	.	.	I	I	.	E	T	.	.	L	.	R
**5**	1	R	I	.	I	S	.	.	.	F	I	.	I	H	.	C	V	I	I	S	.	.	D	S	G	D	.	.	.	.	.	.	.	.	.	.	N	K	A	R	.	R	H	.	Y	K	.	K	Y	.	H	K	.	.	I	I	.	E	T	.	.	L	.	R
**6**	1	R	I	.	I	S	.	.	.	.	I	F	I	H	.	C	.	I	I	S	.	.	D	.	G	D	.	F	P	F	A	.	.	.	.	.	.	.	A	R	.	R	H	.	Y	K	.	.	Y	.	H	K	.	.	I	I	.	E	.	.	.	L	.	R
**7**	1	R	I	.	.	.	.	.	.	F	I	F	I	H	.	C	V	I	I	d	.	.	D	.	G	D	.	F	P	F	A	N	S	N	K	.	N	K	A	R	.	R	H	.	Y	K	I	K	Y	K	H	K	F	.	I	I	.	E	T	.	.	L	.	R
**8**	1	R	I	.	.	S	.	.	.	F	I	F	I	H	.	C	V	I	I	S	.	.	D	.	G	D	.	F	P	F	A	.	.	N	.	.	N	K	A	R	.	R	H	.	Y	K	I	K	Y	.	H	K	.	.	I	I	.	.	.	.	.	L	.	R
**9**	1	R	I	.	.	S	.	.	.	F	I	F	I	H	.	C	V	I	.	.	.	.	D	.	G	D	.	F	P	F	A	.	.	.	K	.	N	K	A	R	.	R	.	.	Y	K	.	.	.	.	H	K	.	.	I	I	.	.	T	I	.	L	.	R
**10**	1	R	I	.	.	S	.	.	.	F	I	F	I	H	.	C	V	.	I	S	.	.	D	.	G	D	.	F	P	F	A	N	S	N	K	.	N	K	A	R	S	R	H	.	Y	K	.	K	Y	.	H	K	.	.	I	I	.	E	T	I	.	L	.	R
**11**	1	R	I	.	.	S	.	.	.	F	I	F	I	.	.	.	V	I	I	S	.	.	D	.	G	D	.	F	P	F	A	N	S	N	.	.	.	.	.	.	.	.	.	.	Y	K	.	K	Y	.	H	K	.	.	I	I	.	E	T	.	.	L	F	R
**12**	1	R	I	.	.	S	.	.	.	F	I	.	I	H	.	C	V	I	I	S	.	.	D	.	G	.	.	.	.	.	.	N	S	N	K	.	N	K	A	R	.	R	H	.	Y	.	.	K	Y	.	H	K	.	A	I	I	.	E	T	.	.	L	.	R
**13**	1	R	I	.	.	S	.	.	.	F	I	.	I	H	.	C	V	I	I	S	.	.	.	.	G	.	.	.	.	.	.	N	S	N	K	.	N	K	A	R	.	R	H	.	Y	K	.	K	Y	.	H	K	.	A	I	I	.	E	T	.	.	L	.	R
**14**	1	R	I	.	.	S	.	.	.	F	I	.	I	H	.	C	V	.	I	S	.	.	D	.	G	.	.	F	P	F	A	N	S	N	K	.	.	K	A	R	.	R	H	.	Y	K	.	K	Y	.	H	K	.	A	I	I	.	E	T	.	.	L	.	R
**15**	1	R	I	.	.	S	.	.	.	F	.	.	.	H	.	C	V	I	I	.	.	.	.	.	.	D	.	F	P	F	A	N	S	N	K	.	N	K	A	R	.	R	H	.	Y	K	.	K	Y	.	H	K	.	.	I	I	.	E	T	.	.	L	F	R
**16**	1	R	I	.	.	S	.	T	.	.	I	F	I	H	.	C	V	I	I	S	.	.	D	.	G	D	.	F	P	F	A	N	S	N	.	.	N	K	.	.	.	.	.	.	Y	K	.	K	Y	.	H	K	.	.	I	I	.	E	T	.	.	L	.	R
**17**	1	R	I	.	.	S	.	T	.	.	I	F	I	H	.	C	V	.	I	S	.	.	D	.	G	D	.	F	P	F	A	N	S	N	.	.	.	.	.	.	.	.	.	.	Y	K	.	K	Y	.	H	K	.	.	I	I	.	E	T	.	.	L	.	.
**18**	1	R	.	.	.	S	.	.	.	.	I	F	I	H	.	C	.	I	I	S	.	.	.	.	G	.	.	.	.	.	.	.	.	.	.	.	.	.	.	.	.	.	.	.	Y	K	.	K	Y	.	H	K	.	A	I	I	.	E	T	.	.	L	.	R
**19**	1	.	I	.	I	S	.	.	.	F	I	F	I	H	.	C	V	I	I	S	.	.	D	S	G	D	.	F	P	F	A	N	S	N	K	.	N	K	A	R	.	R	H	.	Y	K	.	K	Y	.	H	K	.	.	I	I	.	E	T	.	.	.	.	R
**20**	1	.	.	.	.	.	I	.	T	.	.	.	I	H	V	.	V	.	.	.	V	I	D	.	.	.	.	.	.	.	.	.	.	.	.	.	.	.	.	.	.	.	.	K	Y	K	.	K	Y	K	H	K	F	A	.	I	G	E	T	.	.	L	.	.
**21**	1	.	.	R	I	.	I	.	T	.	.	.	I	H	V	.	V	.	.	.	V	I	D	I	.	D	T	F	P	.	.	.	.	N	K	S	N	K	A	R	S	R	H	K	Y	K	.	K	Y	K	H	K	F	.	.	I	G	E	T	.	.	L	.	.
**22**	1	.	.	R	I	.	I	.	T	.	.	.	I	.	V	.	V	.	.	.	V	I	D	I	.	D	K	L	P	F	.	.	.	.	K	.	N	K	A	R	S	R	H	K	Y	K	.	K	Y	K	H	K	F	.	.	I	.	E	T	.	.	L	.	.
**23**	1	.	.	R	I	.	.	.	T	.	.	.	I	H	V	.	.	.	.	.	V	I	D	I	.	D	.	L	P	F	.	.	.	N	K	S	N	K	A	R	S	R	H	K	Y	K	.	.	Y	K	.	K	F	.	.	I	G	E	T	.	.	L	.	.
**24**	1	.	.	R	.	.	.	.	T	.	.	.	I	H	V	.	.	.	.	.	V	I	D	I	.	D	.	L	P	F	.	.	.	N	K	S	N	K	A	.	S	R	H	K	Y	K	.	K	Y	K	H	K	F	.	.	I	G	E	T	.	L	L	.	.

© 2023 Zhao LP et al. *JAMA Network Open.*

eTable 21. Four Mixture Variants with Omicron-Zeta

Four recombinants of Omicron with A) Delta, B) Alpha, C) Zeta and D) Epsilon variants, in which all observed recombinants include polymutants in their respective variant-specific core haplotypes and indicator of 1 and 2 corresponded to the variant (1) recombinaned with Omicron (2)

Epsilon-Omicron	Freq	NSP1 S135	NSP2 T85	NSP3 T24	NSP3 K38	NSP3 G489	NSP3 L1266	NSP3 A1892	NSP4 L264	NSP4 T327	NSP4 L438	NSP4 T492	NSP5 P132	NSP6 I189	NSP9 I65	NSP13 R392	NSP14 I42	NSP15 T112
**Indicator**		2	1	2	2	2	2	2	2	2	2	2	2	2	1	2	2	2
**1**	96	R	.	I	.	S	.	.	F	I	F	I	H	.	.	C	V	I
**2**	94	R	I	I	.	S	.	.	F	I	F	I	H	.	.	C	V	I
**3**	88	R	.	I	.	S	.	.	F	I	F	I	H	.	.	C	V	I
**4**	8	R	I	I	.	S	.	.	F	I	F	I	H	.	.	C	V	I
**5**	8	R	.	I	.	S	.	.	F	I	F	I	H	.	.	C	V	.
**6**	7	R	.	I	.	S	.	.	F	I	F	I	H	.	.	C	V	.
**7**	6	R	.	I	.	S	.	.	F	I	F	I	H	.	.	C	V	I
**8**	6	R	.	I	.	S	.	.	F	I	F	I	H	.	.	C	V	I
**9**	6	R	.	I	.	S	.	.	F	I	F	I	H	.	.	C	V	I
**10**	6	R	.	I	.	S	.	.	F	I	F	I	H	.	V	C	V	I
**11**	5	R	I	I	.	S	.	.	F	I	F	I	H	.	.	C	V	I
**12**	5	R	.	I	.	S	.	.	F	I	F	I	H	.	.	C	V	I
**13**	4	R	.	I	.	S	.	.	F	I	F	I	H	.	.	C	V	I
**14**	4	R	.	I	.	S	.	.	F	I	F	I	H	.	.	C	V	I
**15**	4	.	I	.	R	.	I	T	.	.	.	I	H	V	.	.	V	.
**16**	3	R	I	I	.	S	.	.	F	I	F	I	H	.	.	C	V	I
**17**	3	R	I	I	.	S	.	.	F	I	F	I	H	.	.	C	V	I
**18**	3	R	I	I	.	S	.	.	F	I	F	I	H	.	.	C	V	I
**19**	3	R	.	I	.	S	.	.	F	I	F	I	H	.	.	C	V	I
**20**	3	R	.	I	.	S	.	.	F	I	F	I	H	.	.	C	V	I

Epsilon-Omicron	Spike T19	Spike A27	Spike A67	Spike T95	Spike G142	Spike L212	Spike V213	Spike G339	Spike R346	Spike S371	Spike S373	Spike S375	Spike T376	Spike D405	Spike R408	Spike K417	Spike N440	Spike G446	Spike L452
**Indicator**	2	2	2	2	2	2	2	2	2	2	2	2	2	2	2	2	2	2	1
**1**	I	S	.	.	D	.	G	D	.	F	P	F	A	N	S	N	K	.	.
**2**	I	S	.	.	D	.	G	D	.	F	P	F	A	N	S	N	K	.	.
**3**	I	S	.	.	D	.	G	D	.	F	P	F	A	N	S	N	K	.	.
**4**	I	S	.	.	D	.	G	D	.	F	P	F	A	N	S	N	K	.	.
**5**	I	S	.	.	D	.	G	D	.	F	P	F	A	N	S	N	K	.	.
**6**	I	S	.	.	D	.	G	D	.	F	P	F	A	N	S	N	K	.	.
**7**	I	S	.	.	D	.	G	D	.	F	P	F	A	N	S	N	.	.	.
**8**	I	S	.	.	D	.	G	D	.	F	P	F	A	N	S	N	.	.	.
**9**	I	S	.	.	D	.	G	.	.	.	.	.	.	N	S	N	K	.	.
**10**	I	S	.	.	D	.	G	D	.	F	P	F	A	N	S	N	K	.	.
**11**	I	S	.	.	D	.	G	D	.	F	P	F	A	N	S	N	K	.	.
**12**	I	S	.	.	D	.	G	D	.	F	P	F	A	N	S	N	K	.	.
**13**	I	S	.	.	D	.	G	D	.	F	P	F	A	N	S	N	K	.	.
**14**	I	S	.	.	D	.	G	D	.	F	P	F	A	N	S	N	K	.	.
**15**	.	.	V	I	D	I	.	D	K	L	P	F	.	.	.	N	K	S	.
**16**	I	S	.	.	D	.	G	D	.	F	P	F	A	N	S	.	K	.	.
**17**	I	S	.	.	D	.	G	D	.	F	P	F	A	N	S	.	.	.	.
**18**	I	S	.	.	D	.	G	D	.	F	P	F	A	N	S	N	.	.	.
**19**	I	.	.	.	D	.	G	D	.	F	P	F	A	N	S	N	K	.	.
**20**	I	S	.	.	D	.	G	D	.	F	P	F	A	N	S	.	.	.	.

Epsilon-Omicron	Spike S477	Spike T478	Spike E484	Spike Q493	Spike G496	Spike Q498	Spike N501	Spike Y505	Spike T547	Spike H655	Spike N679	Spike P681	Spike N764	Spike D796	Spike N856	Spike Q954	Spike N969	Spike L981	NS3 Q57	NS3 T223	E T9	M D3	M Q19	M A63	N P13	N R203	N G204	N T205	N S413
**Indicator**	2	2	2	2	2	2	2	2	2	2	2	2	2	2	2	2	2	2	1	2	2	2	2	2	2	2	2	1	2
**1**	N	K	A	R	.	R	Y	H	.	Y	K	H	K	Y	.	H	K	.	H	I	I	.	E	T	L	K	R	.	R
**2**	N	K	A	R	.	R	Y	H	.	Y	K	H	K	Y	.	H	K	.	.	I	I	.	E	T	L	K	R	.	R
**3**	N	K	A	R	.	R	Y	H	.	Y	K	H	K	Y	.	H	K	.	.	I	I	.	E	T	L	K	R	I	R
**4**	N	K	A	R	.	R	Y	H	.	Y	K	H	K	Y	.	H	K	.	.	I	I	.	E	T	L	.	.	.	R
**5**	N	K	A	R	.	R	Y	H	.	Y	K	H	K	Y	.	H	K	.	H	I	I	.	E	T	L	K	R	.	R
**6**	N	K	A	R	.	R	Y	H	.	Y	K	H	K	Y	.	H	K	.	.	I	I	.	E	T	L	K	R	I	R
**7**	N	K	A	R	.	R	Y	H	.	Y	K	H	K	Y	.	H	K	.	H	I	I	.	E	T	L	K	R	.	R
**8**	N	K	A	R	.	R	Y	H	.	Y	K	H	K	Y	.	H	K	.	.	I	I	.	E	T	L	K	R	I	R
**9**	N	K	A	R	.	R	Y	H	.	Y	K	H	K	Y	.	H	K	.	H	I	I	.	E	T	L	K	R	.	R
**10**	N	K	A	R	.	R	Y	H	.	Y	K	H	K	Y	.	H	K	.	.	I	I	.	E	T	L	K	R	.	R
**11**	N	K	A	R	.	.	.	.	.	Y	K	H	K	Y	.	H	K	.	.	I	I	.	E	T	L	K	R	.	R
**12**	N	K	A	R	.	.	.	.	.	Y	K	H	K	Y	.	H	K	.	.	I	I	.	E	T	L	K	R	I	R
**13**	N	K	A	R	.	R	Y	H	.	Y	K	H	K	Y	.	H	K	.	H	I	I	.	Z	T	L	K	R	.	R
**14**	N	K	A	R	.	R	Y	H	.	Y	K	H	K	Y	.	H	K	.	.	I	I	.	E	T	.	K	R	I	R
**15**	N	K	A	R	S	R	Y	H	K	Y	K	H	K	Y	K	H	K	F	.	.	I	G	E	T	L	K	R	.	.
**16**	N	K	A	R	.	R	Y	H	.	Y	K	H	K	Y	.	H	K	.	.	I	I	.	E	T	L	K	R	.	R
**17**	N	K	A	R	.	R	Y	H	.	Y	K	H	K	Y	.	H	K	.	.	I	I	.	E	T	L	K	R	.	R
**18**	.	.	.	.	.	.	.	.	.	Y	K	H	K	Y	.	H	K	.	.	I	I	.	E	T	L	K	R	.	R
**19**	N	K	A	R	.	R	Y	H	.	Y	K	H	K	Y	.	H	K	.	H	I	I	.	E	T	L	K	R	.	R
**20**	N	K	A	R	.	R	Y	H	.	Y	K	H	K	Y	.	H	K	.	H	I	I	.	E	T	L	K	R	.	R

© 2023 Zhao LP et al. *JAMA Network Open.*

eTable 22. Four Mixture Variants with Omicron-Epsilon

Geo- and temporal distribution of Epsilon-Omicron recombinants in the world.

Collection Date	Africa/Mauritius	Africa/South Africa	Asia/Israel	Asia/Japan	Asia/Malaysia	Asia/Singapore	Asia/South Korea	Asia/Thailand	Europe/Austria	Europe/Belgium	Europe/Croatia	Europe/Czech Republic	Europe/Denmark	Europe/Estonia	Europe/Finland	Europe/France	Europe/Germany	Europe/Ireland	Europe/Italy	Europe/Lithuania	Europe/Luxembourg	Europe/Netherlands	Europe/Norway	Europe/Poland	Europe/Portugal	Europe/Slovakia	Europe/Slovenia	Europe/Spain	Europe/Sweden	Europe/Switzerland	Europe/Turkey	Europe/United Kingdom	North America/Canada	North America/Costa Rica	North America/USA	Oceania/Australia	Oceania/New Zealand	South America/Brazil	South America/Chile	South America/Colombia	total
2022-03-15								1																	1				1			1	1		1						6
2022-03-16			1													1												1				1	1		2			1			8
2022-03-17			1													1						1		1					2			1	1								8
2022-03-18																	1	1										1	1			3	1		2						10
2022-03-19													2			1																1			1		1				6
2022-03-20																																			1						1
2022-03-21																2											1					3	1		1						8
2022-03-22									1			1	1			1	1	4										1				2			3						15
2022-03-23					1																			2								3			2						8
2022-03-24								1													1		2									5			3	1					13
2022-03-25																			1													1			1						3
2022-03-26				3	3																																				6
2022-03-27	1											1																				1									3
2022-03-28														1	1	1	1											1	1			1	1			1					9
2022-03-29				2						1							2											1				1	3								10
2022-03-30													1			1												2							1						5
2022-03-31											1	1					3											2				1									8
2022-04-01												1																				3			1				1		6
2022-04-02		1	1														1															3	1								7
2022-04-03																	1										1						2		2						6
2022-04-04				1												2	1																		1				1		6
2022-04-05											1							1					1					1				1			1		1			1	8
2022-04-06		1									3																	1	1			7			2						15
2022-04-07			1														1					1		1	1						1	3		1	1						11
2022-04-08			2												1														1			4									8
2022-04-09																			1													4	2		3						10
2022-04-10							1					1										2	1									3			1						9
2022-04-11								2				1	6			5	3				1				2							7	1		2	1					31
2022-04-12												2	2				3			1			1					1				5	1		1						17
2022-04-13		1	1									2					2						1							1		3	1		1	1					14
2022-04-14																	3					6						1	1			7	1		4						23
2022-04-15																	4															1	1		4						10
2022-04-16			1							1	1					1	4															2	1		3	1					15
2022-04-17							1										1									1						3			2						8
2022-04-18			1														4															9	1		3	2					20
2022-04-19			1										8			1	3				1	1										7	1		3	1			1		28
2022-04-20								1									1					1						2				3	1		1						10
2022-04-21			1										2				3		1			1							1			3	1		5						18
2022-04-22			2				1			1		1	2				4												2			8			5						26
2022-04-23							1					1	1				2		1													5			4						15
2022-04-24										1			1				3															1	1		7						14
2022-04-25										1			2			2	6									1						2	2		7						23
2022-04-26										2			1				3				1	1							1			9			14						32
2022-04-27						1				1			1				1															6	2		4						16
2022-04-28																	5												1			8			5						19
2022-04-29																	1															5	1		8						15
2022-04-30																																1			3		1				5
2022-05-01										1			1																			1	1		2						6
2022-05-02										2			1						1													1			3						8
2022-05-03																	1											3				4			3						11
2022-05-04													1				1		2													2									6
2022-05-05																	1																								1
2022-05-06																		1														2									3
2022-05-08										1																															1
2022-05-09										1																															1
total	1	3	13	6	4	1	4	5	1	13	6	12	33	1	2	19	71	7	7	1	4	14	6	4	4	2	2	18	13	1	1	158	31	1	124	8	3	1	3	1	609

© 2023 Zhao LP et al. *JAMA Network Open.*

eTable 23. Mixture Variant and Corresponding Lineages

Relationship of mixture variants with their assigned lineages

**Lineage**	**Omicron-Delta**	**Omicron-Epsilon**	**Omicron-Alpha**	**Omicron-Zeta**	**Alpha-Epsilon**	**Delta-Kappa**	**Delta-Zeta**	total
AY.25						1		1
AY.38							1	1
AY.4.14						1		1
AY.4.15						1		1
AY.45							1	1
BA.1	1	1	3	1				6
BA.1.1		30	2	2				34
BA.1.1.1		6						6
BA.1.1.14		1						1
BA.1.1.15		1						1
BA.1.1.2		5						5
BA.1.14		3						3
BA.1.15		1						1
BA.1.17		1						1
BA.1.17.2		1						1
BA.1.18		2						2
BA.1.19		1						1
BA.2	2	354	15	5	6			382
BA.2.1		5						5
BA.2.10		5		1	1			7
BA.2.12		4						4
BA.2.15		2						2
BA.2.2		1						1
BA.2.20		3						3
BA.2.21		1						1
BA.2.22		3						3
BA.2.23		4						4
BA.2.24		1						1
BA.2.26		1						1
BA.2.3		88			3			91
BA.2.3.4		9						9
BA.2.4		1						1
BA.2.7		1						1
BA.2.8		1						1
BA.2.9		71	5					76
BA.4		1						1
BA.5				1				1
total	3	609	25	10	10	3	2	662

© 2023 Zhao LP et al. *JAMA Network Open.*

eTable 24. Unidentifiable Omicron Viruses and Corresponding Lineages

HAI-unpredictable Omicron viruses and their assigned lineages

	**O1**	**O2**	**O3**	**O4**	total
BA.1	22	5	55	3	85
BA.1.1	3		54	1	58
BA.1.1.1			4		4
BA.1.1.13			1		1
BA.1.1.18			1		1
BA.1.15			2		2
BA.1.15.2			1		1
BA.1.17			5		5
BA.1.20			1		1
BA.1.9			1		1
BA.2	510	253		323	1086
BA.2.10	9	5			14
BA.2.12	23	13			36
BA.2.12.1	18	6			24
BA.2.18	2				2
BA.2.19		1			1
BA.2.23	35	9		3	47
BA.2.3	18	19		1	38
BA.2.3.2		3			3
BA.2.3.3	1				1
BA.2.31	4	2			6
BA.2.32	1				1
BA.2.5	1	1			2
BA.2.6	2				2
BA.2.7	2				2
BA.2.8		2			2
BA.2.9	27	16		9	52
BA.3	1	1			2
BA.3.1	4				4
BA.4	1				1
BA.5	3				3
Unassigned	131	62	9		202
XE	8	1			9
total	826	399	134	340	1699

© 2023 Zhao LP et al. *JAMA Network Open.*

eTable 25. New Mutations Among Variant-Unassigned and Unidentifiable Viruses

Ab initio mutations (excluding all core polymutants of 14 known variants) have been foud to significant temporal trend

	Polymutant	cluster	p-vlaue	LAMP	LAMP-max
1	NSP1_F143	O1	3.43E-02	2.25E-02	7.62E-02
2	NSP1_K141	O1	3.43E-02	2.25E-02	7.62E-02
3	NSP1_S142	O1	3.43E-02	2.25E-02	7.62E-02
4	Spike_A684	O1	1.94E-02	2.00E-02	9.05E-02
5	Spike_I68	O1	4.10E-02	2.09E-02	1.22E-01
6	NSP2_F356	O2	1.04E-02	2.34E-02	1.09E-01
7	NSP6_L105	O2	2.76E-04	8.18E-02	2.09E-01
8	NS3_H78	O3	3.56E-02	9.53E-02	1.67E-01
9	Spike_L24	O4	3.12E-05	3.30E-01	5.33E-01
10	Spike_P25	O4	1.66E-05	3.32E-01	5.41E-01
11	NSP6_F108	O5	2.09E-06	4.42E-01	6.02E-01
12	NSP6_G107	O5	4.32E-05	5.20E-01	6.70E-01
13	NSP6_S106	O5	6.73E-05	5.14E-01	6.61E-01
14	N_E31	O6	1.99E-02	6.52E-01	7.15E-01
15	N_R32	O6	1.70E-02	6.39E-01	7.00E-01
16	N_S33	O6	1.03E-02	6.49E-01	7.18E-01

© 2023 Zhao LP et al. *JAMA Network Open.*

eFigure 1. Heatmap-representation of selected polymutant temporal profile from January 1, 2020 to March 14, 2022 within every variant: A) Alpha, B) Beta, C) Delta, D) Epsilon, E) Eta, F) Gamma, G) GH/490R, H) Iota, I) Kappa, J) Lambda, K) Mu L) Omicron, M) Theta, N) Zeta and O) variant-unassigned viruses. In each profile, estimated locally-averaged mutation percentages (LAMP) take value from 0 to 1, and are color-coded (the legend), and each row corresponds to a polymutant while each column corresponds to collection date.

A)

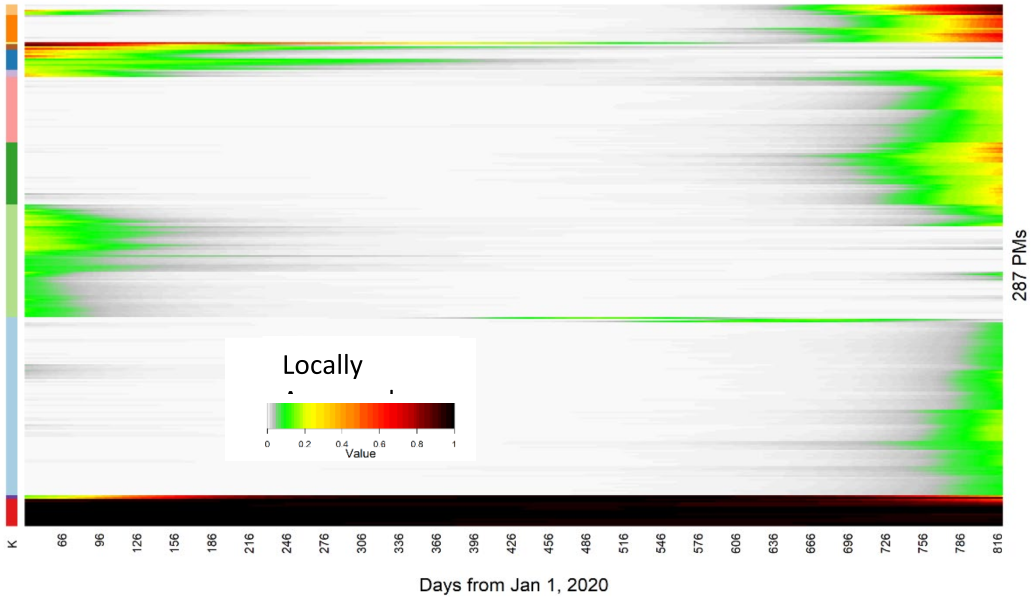

© 2023 Zhao LP et al. *JAMA Network Open.*

B) Beta

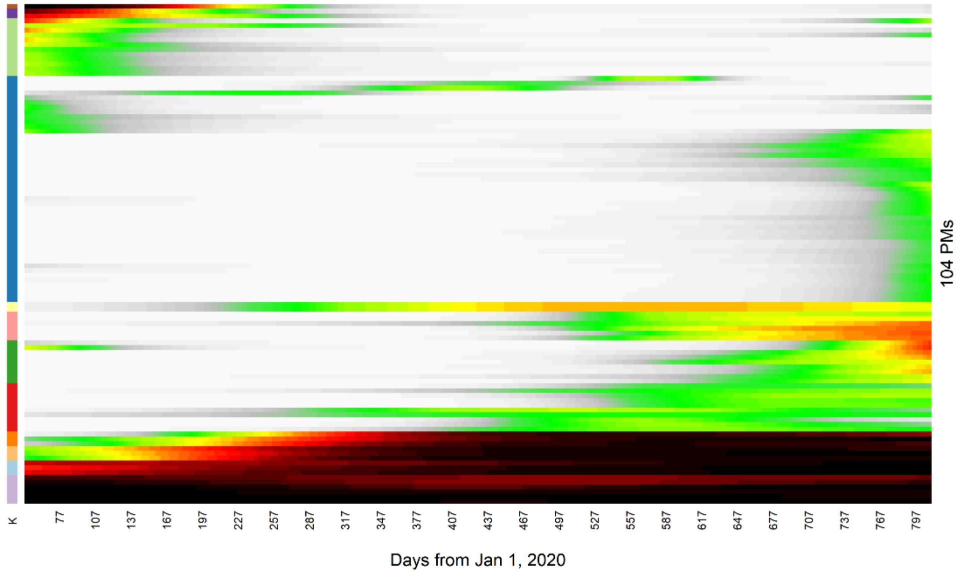

© 2023 Zhao LP et al. *JAMA Network Open.*

C) Delta

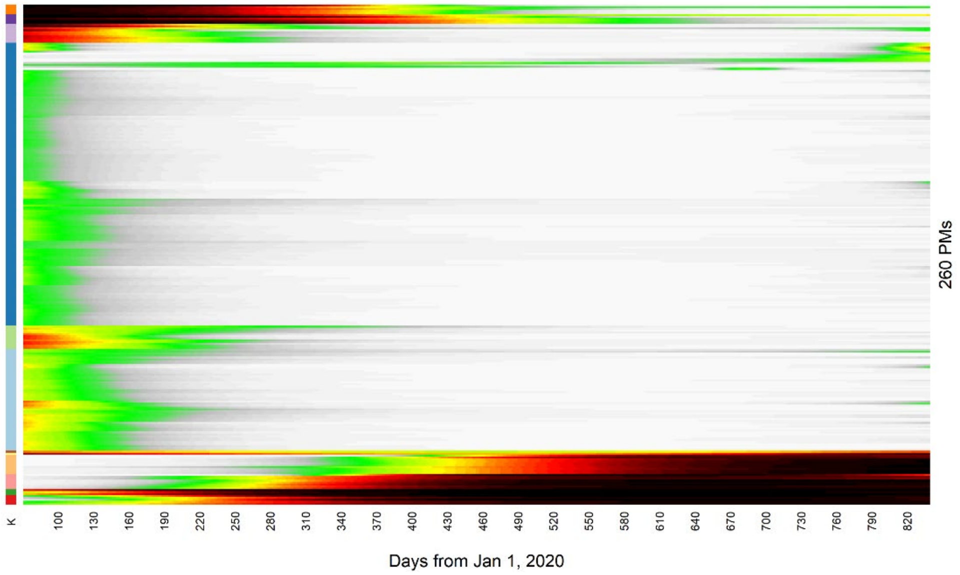

© 2023 Zhao LP et al. *JAMA Network Open.*

D) Epsilon

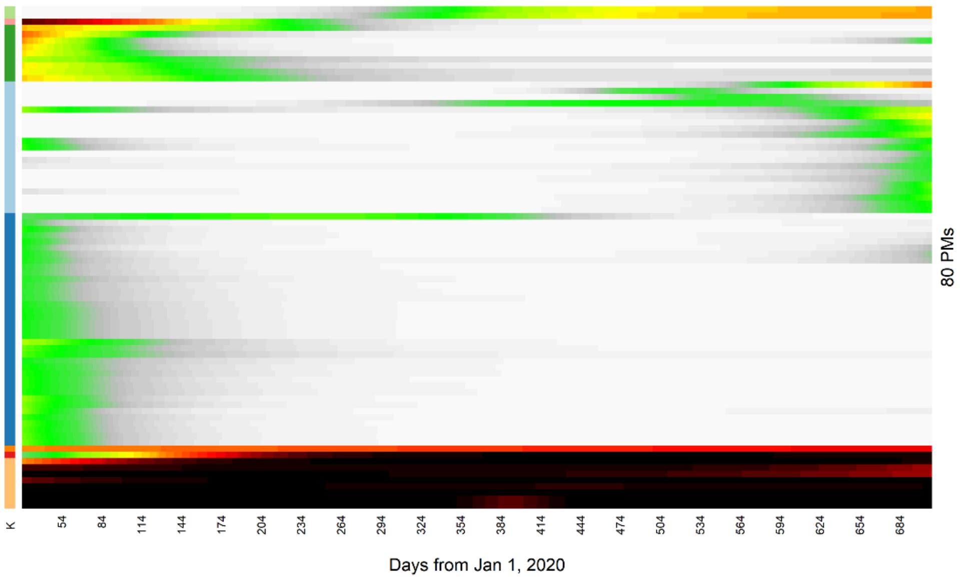

© 2023 Zhao LP et al. *JAMA Network Open.*

E) Eta

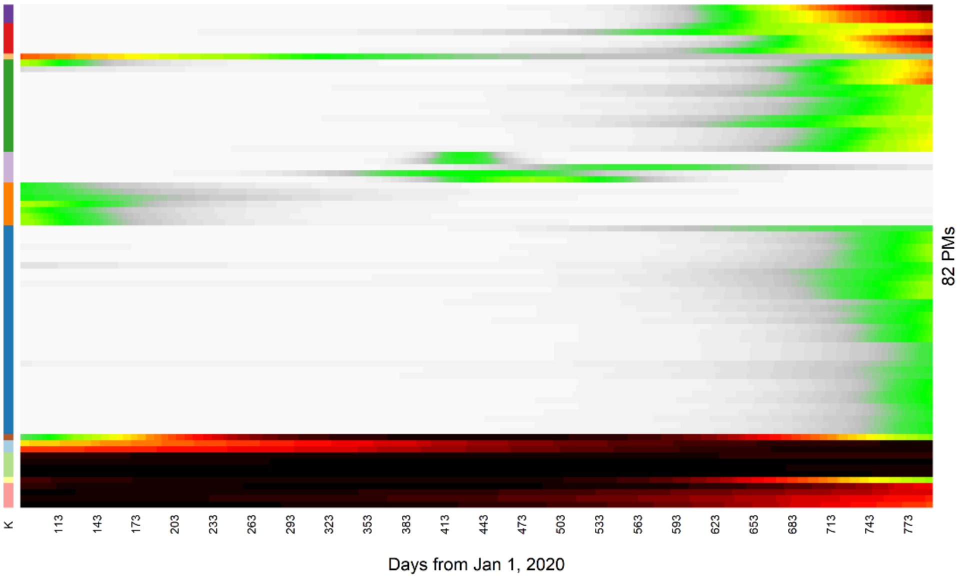

© 2023 Zhao LP et al. *JAMA Network Open.*

F) Gamma

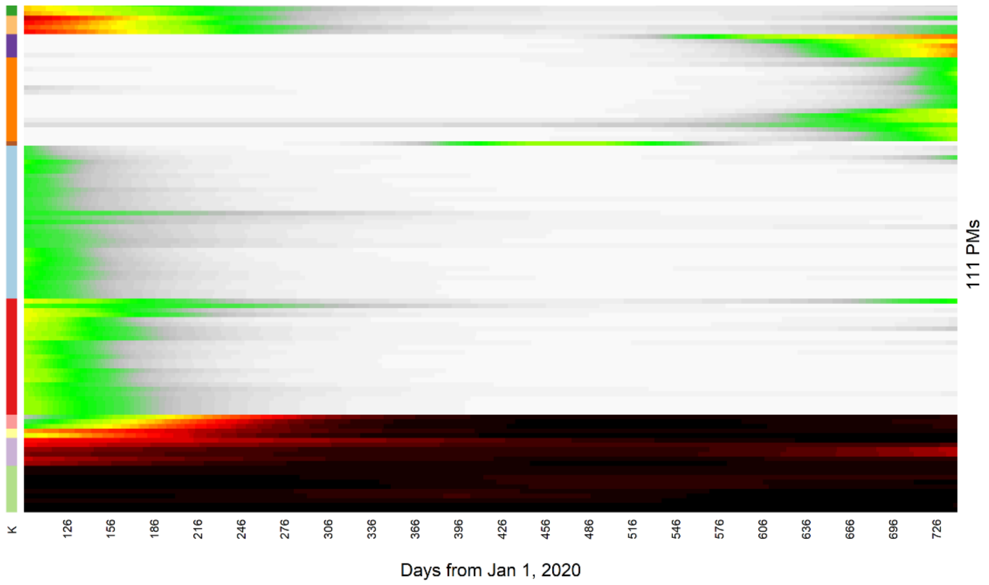

© 2023 Zhao LP et al. *JAMA Network Open.*

G) GH/450R

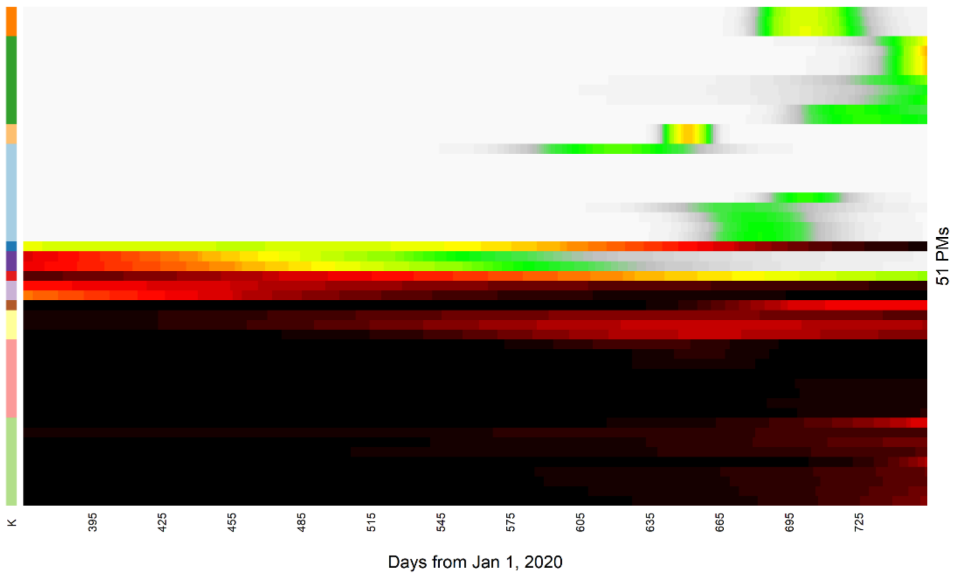

© 2023 Zhao LP et al. *JAMA Network Open.*

H) Iota

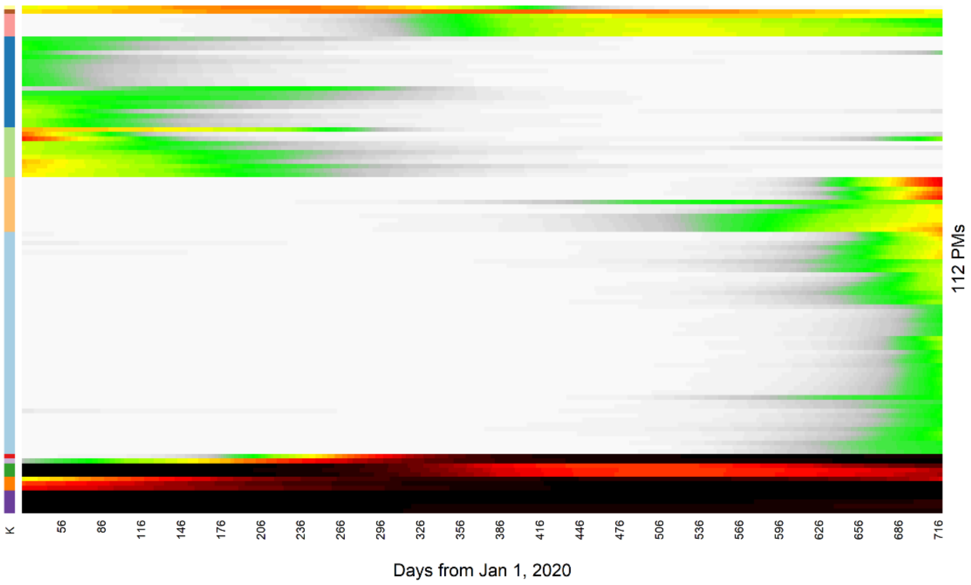

© 2023 Zhao LP et al. *JAMA Network Open.*

I) Kappa

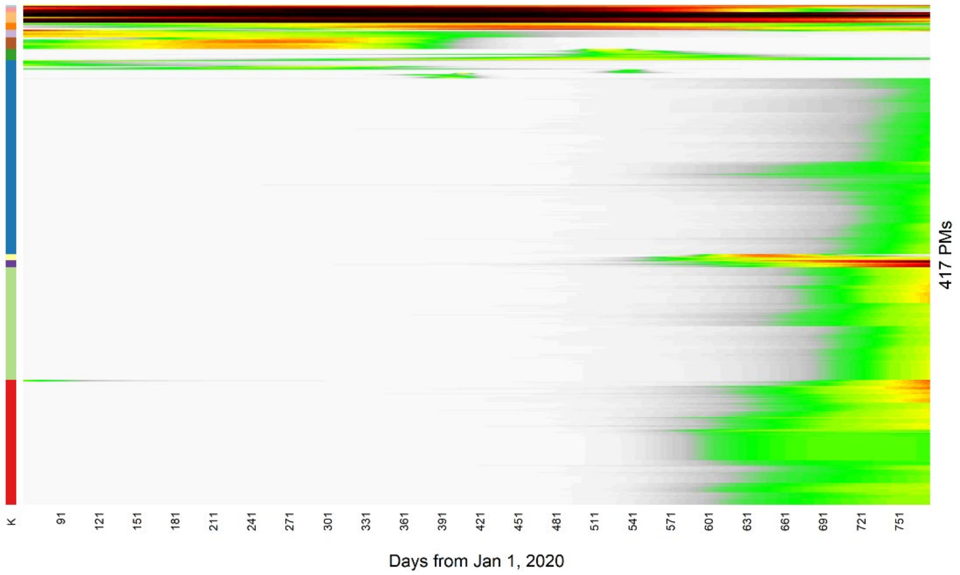

© 2023 Zhao LP et al. *JAMA Network Open.*

J) Lambda

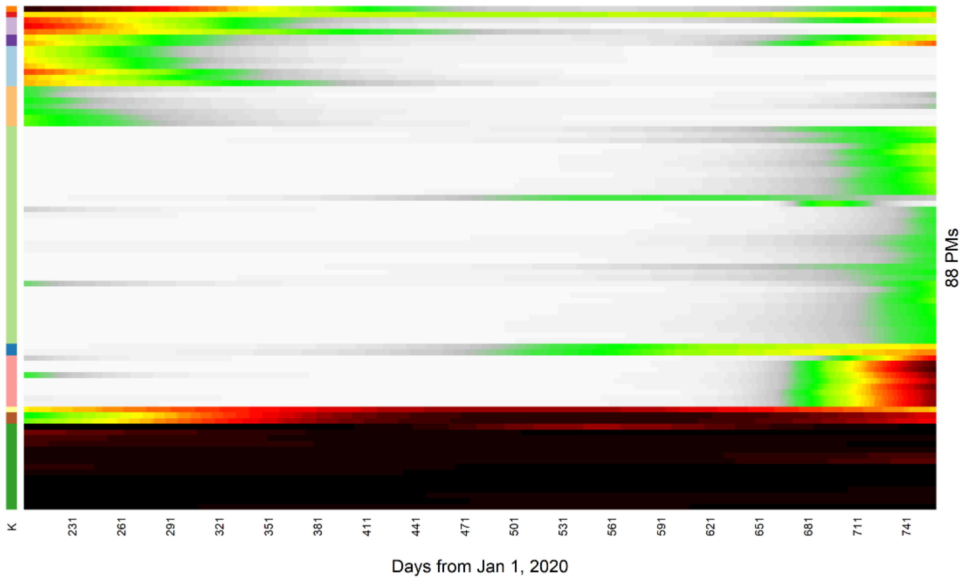

© 2023 Zhao LP et al. *JAMA Network Open.*

L) Omicron

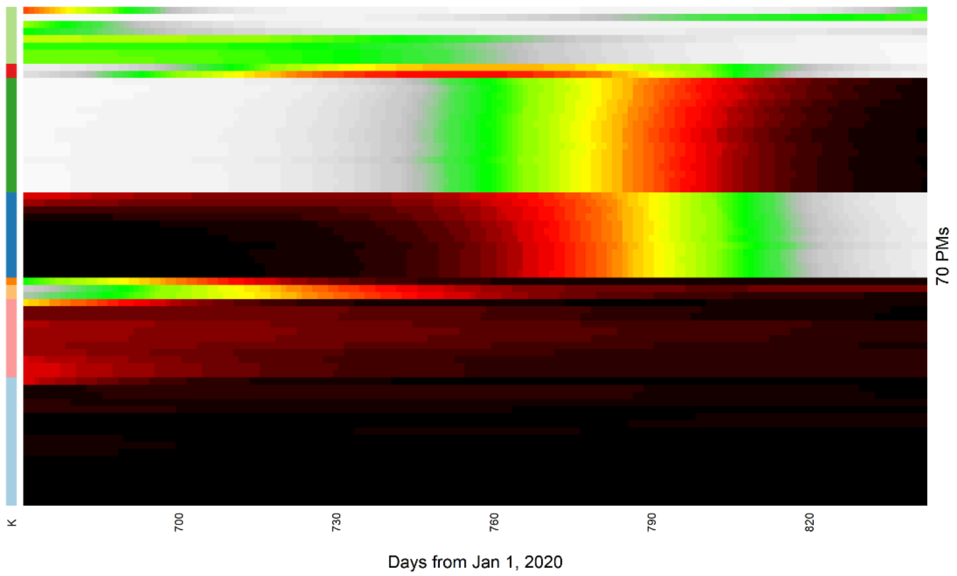

© 2023 Zhao LP et al. *JAMA Network Open.*

M) Mu

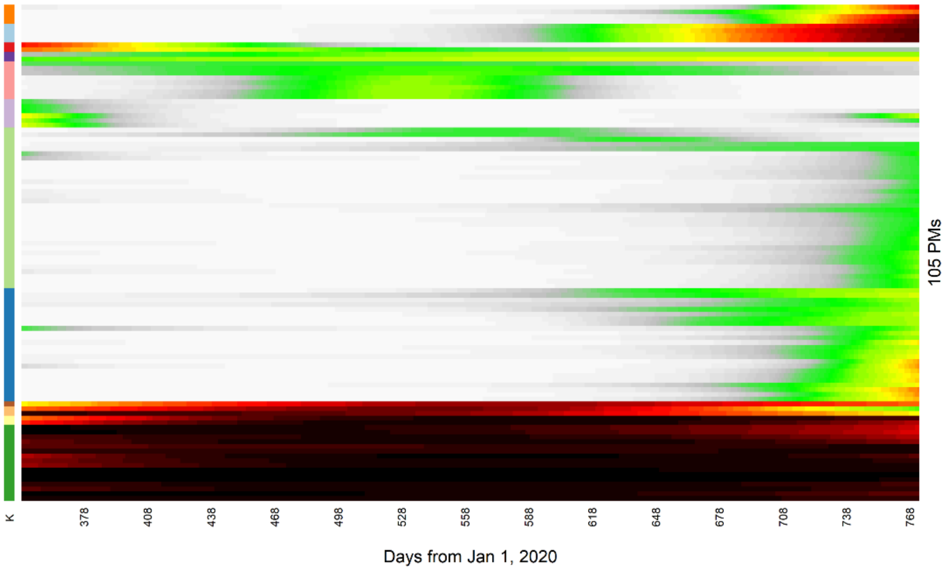

© 2023 Zhao LP et al. *JAMA Network Open.*

N) Theta

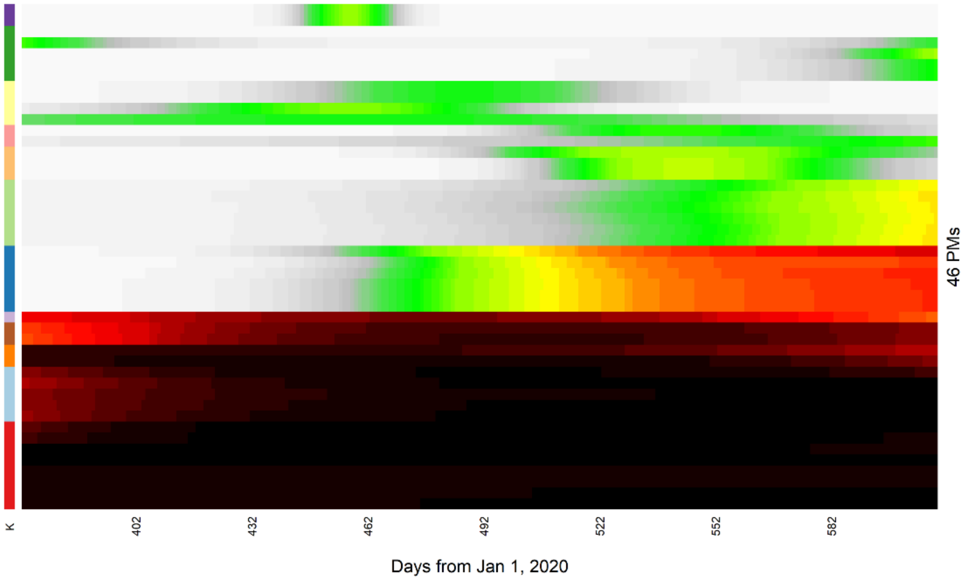

© 2023 Zhao LP et al. *JAMA Network Open.*

N) Zeta

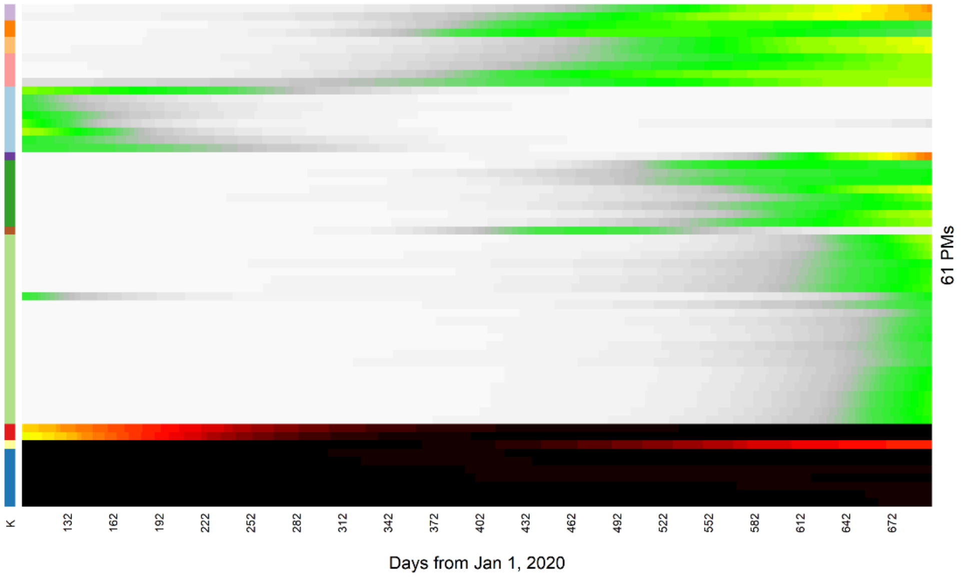

© 2023 Zhao LP et al. *JAMA Network Open.*

O) Unassigned viruses

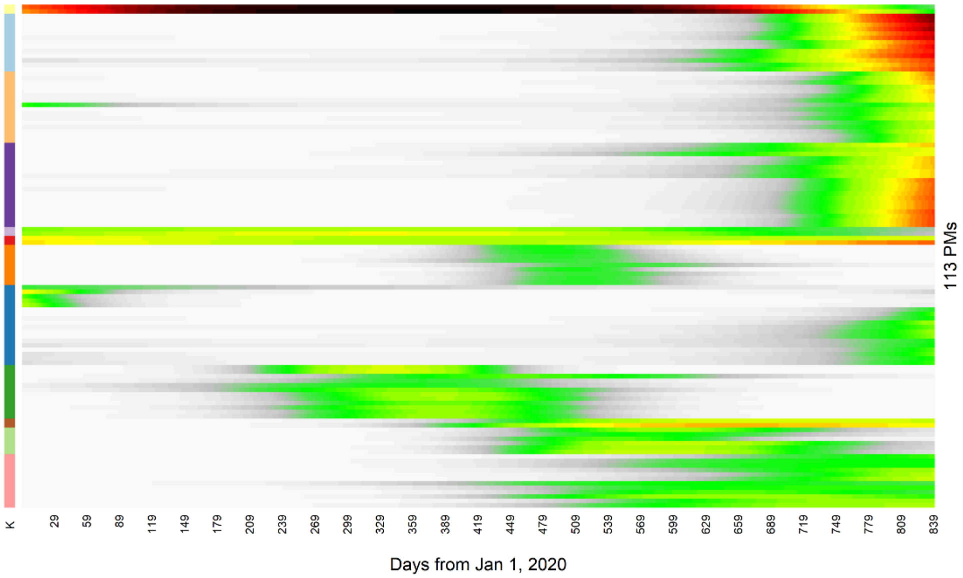

© 2023 Zhao LP et al. *JAMA Network Open.*

eFigure 2. Misclassification errors by haplotype-based variant prediction (HVP), when the prediction probability threshold value is set at 0.9 to 1

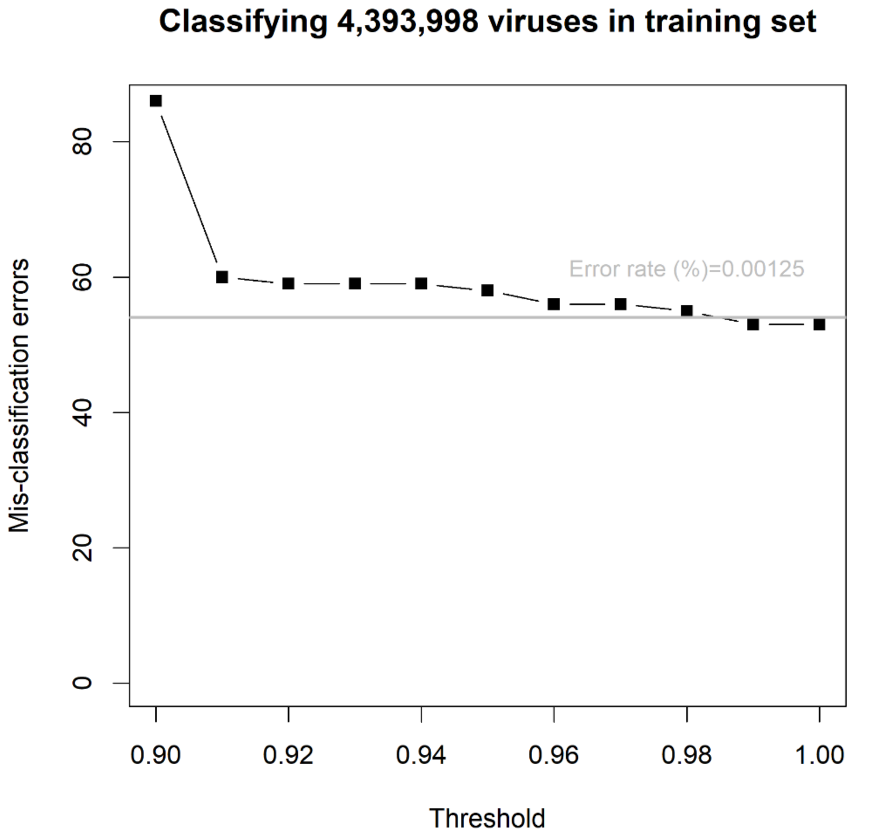

© 2023 Zhao LP et al. *JAMA Network Open.*

eFigure 3. Temporal patterns of sixteen polymutants identified from variant-unassigned 524 viruses that are unpredictable by HAI, excluding those core polymutants of all fourteen variants.

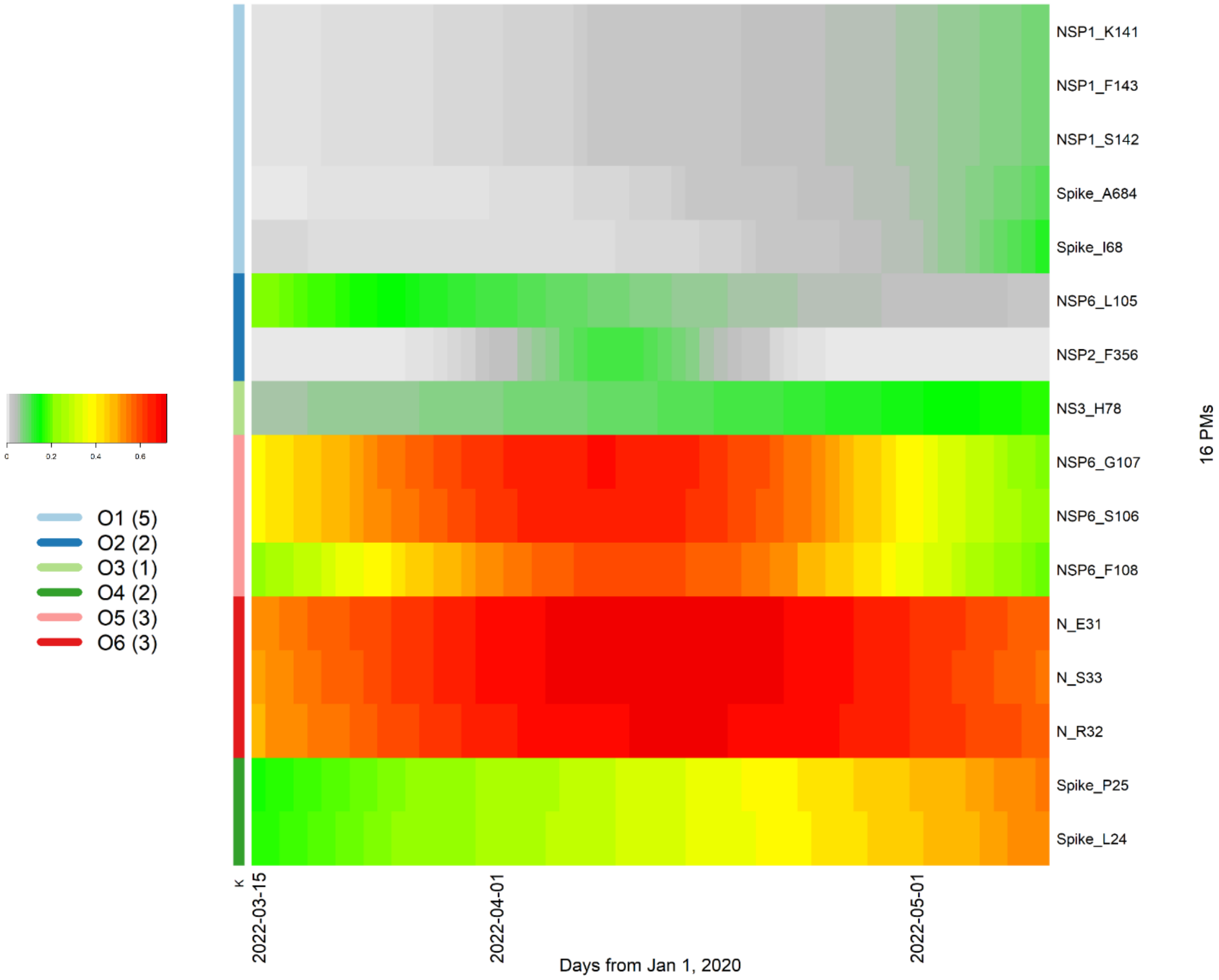

© 2023 Zhao LP et al. *JAMA Network Open.*
