# Supplementary material for: Using Haplotype-Based Artificial Intelligence to Evaluate SARS-CoV-2 Novel Variants and Mutations
Source: JAMA Netw Open. 2023 Feb 21;6(2):e230191. doi: 10.1001/jamanetworkopen.2023.0191 (PMC9945077; doi:10.1001/jamanetworkopen.2023.0191)
Supplement: Supplement 2. — Data Sharing Statement [file jamanetwopen-e230191-s002.pdf]

Data Sharing Statement

Zhao. Using Haplotype-Based Artificial Intelligence to Evaluate SARS-CoV-2 Novel Variants and Mutations. *JAMA Netw Open*. Published online February 21, 2023. doi: 10.1001/jamanetworkopen.2023.0191

Data
Data available: No

© 2023 Zhao LP et al. *JAMA Network Open*.
